# A Selective Pro‐Oxidant Drives Redox Reprogramming to Induce Ferroptosis and Immune Remodeling in Glioblastoma

**DOI:** 10.1002/smmd.70058

**Published:** 2026-10-08

**Authors:** Shilpi Singh, Iteeshree Mohapatra, Katelyn Kim, Raza Ali Naqvi, Devanjan Dey, Laura Mohapatra, Davis Seelig, Afsar R. Naqvi, Amar Singh, Kathleen Boris‐Lawrie, Stefan Kim, Gatikrushna Singh

**Affiliations:** ^1^ Department of Neurosurgery University of Minnesota Minneapolis Minnesota USA; ^2^ Bimeda Biologicals Oakdale Minnesota USA; ^3^ Department of Periodontics, College of Dentistry University of Illinois Chicago Chicago Illinois USA; ^4^ Department of Surgery Schulze Diabetes Institute, University of Minnesota Minneapolis Minnesota USA; ^5^ Department of Veterinary Clinical Sciences, College of Veterinary Medicine University of Minnesota Saint Paul Minnesota USA; ^6^ Department of Veterinary and Biomedical Sciences University of Minnesota Saint Paul Minnesota USA

**Keywords:** anti‐tumor immunity, ferroptosis, glioblastoma, macrophage polarization, oxidative stress, pro‐oxidant, reactive oxygen species

## Abstract

Glioblastoma remains a highly lethal malignancy characterized by profound therapeutic resistance driven, in part, by adaptive redox homeostasis. Although elevated reactive oxygen species support tumor progression, glioblastoma relies on robust antioxidant systems including the *SLC7A11*‐*GPX4* axis to buffer oxidative stress, creating a targetable vulnerability. Here, we investigate *Rosasina*, a sodium‐chlorite (NaClO_2_)‐derived, redox‐active small molecule, as a selective pro‐oxidant strategy to disrupt glioblastoma antioxidant resilience and induce ferroptotic cell death. *Rosasina* preferentially increased intracellular ROS in glioblastoma cells relative to normal human astrocytes, with hydrogen peroxide representing the predominant ROS species. This oxidative shift was accompanied by expansion of the labile iron pool and enhanced lipid peroxidation. In orthotopic glioblastoma models, *Rosasina* was well tolerated, significantly reduced tumor growth, prolonged survival, and exhibited no evidence of treatment‐related systemic toxicity, with preserved histological integrity of major organs. Mechanistically, *Rosasina* induces a coordinated pro‐ferroptotic transcriptional program, characterized by upregulation of iron uptake and lipid peroxidation pathways (*NCOA4, NOX5, ACSL4, TFRC*), alongside suppression of antioxidant defenses, including *GPX4, GCH1, SCD1*, and *SLC7A11*. In addition to its tumor‐intrinsic effects, *Rosasina* promoted macrophage polarization toward an anti‐tumor, pro‐inflammatory M1‐like phenotype without compromising macrophage viability, suggesting a potential role in tumor immune microenvironment reprogramming. Collectively, these findings establish *Rosasina* as a redox‐directed therapeutic that selectively exploits the oxidative vulnerabilities of glioblastoma to induce ferroptosis simultaneously promoting antitumor immune reprogramming. This study provides a mechanistic framework for the development of redox‐targeted therapies for glioblastoma.

## Highlights

- *Rosasina* selectively increases oxidative stress in glioblastoma cells while sparing normal cells.
- *Rosasina* preferentially induces hydrogen peroxide accumulation by suppressing catalase activity.
- *Rosasina* triggers ferroptotic cell death through lipid peroxidation and intracellular iron accumulation.
- *NCOA4* and *NOX5* are critical mediators of *Rosasina*‐induced ferroptosis in glioblastoma.
- *Rosasina* suppressed tumor growth in vivo, prolonged survival, exhibited a favorable preclinical safety profile, and promoted anti‐tumor M1‐like macrophage polarization.

## Graphical abstract

*Rosasina* selectively targets glioblastoma by activating the *NCOA4*‐*NOX5* redox pathway, inducing ferroptosis, and remodeling the tumor immune microenvironment through M1‐like macrophage polarization. These coordinated mechanisms suppress tumor growth and establish *Rosasina* as a promising therapeutic candidate for glioblastoma.

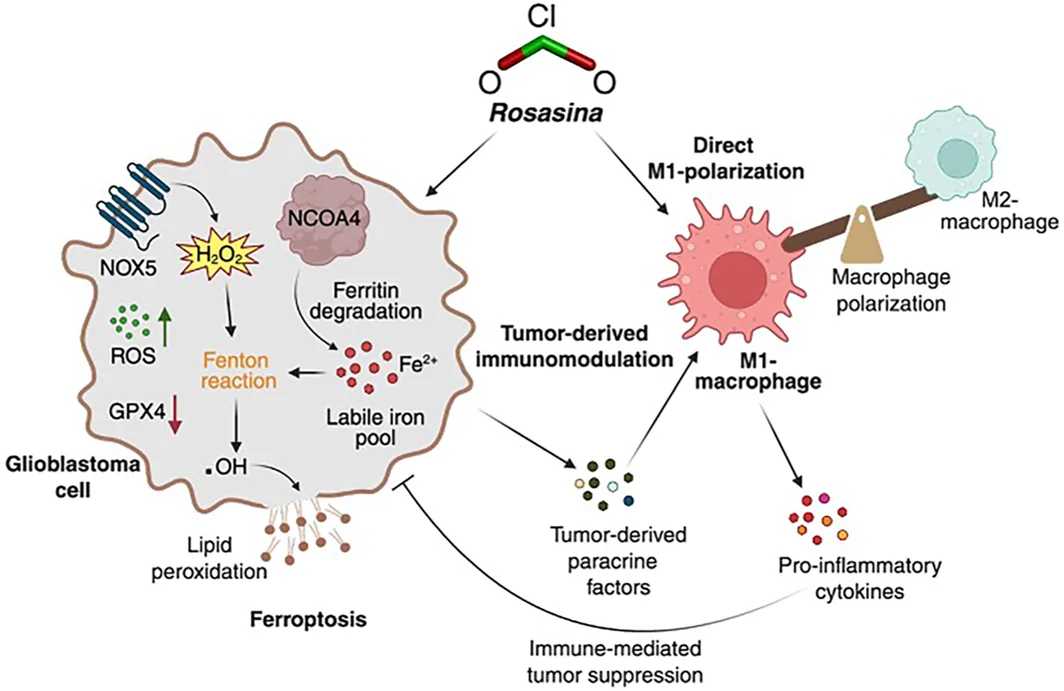

## 1 Introduction

Reactive oxygen species (ROS) are fundamental regulators of cellular physiology, functioning as second messengers in pathways governing proliferation, differentiation, immune activation, and stress adaptation. Under homeostatic conditions, ROS generated by mitochondrial respiration, NADPH oxidases, and peroxisomal metabolism are tightly balanced by antioxidant systems, maintaining redox equilibrium. Disruption of this balance leads to oxidative damage of lipids, proteins, and nucleic acids, ultimately compromising cell viability [1, 2]. In cancer, sustained oxidative stress fuels genomic instability and metabolic adaptation while simultaneously creating a dependence on antioxidant buffering mechanisms, thereby exposing a therapeutically exploitable vulnerability [3, 4, 5, 6].

Glioblastoma, the most aggressive primary brain tumor in adults, exemplifies this redox dependency [7]. Glioblastoma cells sustain elevated basal ROS through oncogenic PI3K‐AKT and MAPK signaling and persistent mitochondrial activity [8, 9], while preserving viability by upregulating antioxidant defense programs including the *SLC7A11*‐*GPX4* axis, and CoQ10 dependent lipid antioxidant signaling. These pathways suppress peroxide accumulation and lipid peroxidation, enabling tumor cells to proliferate under hypoxic and genotoxic stress [10, 11, 12]. Disruption of this redox equilibrium, particularly through excessive hydrogen peroxide (H_2_O_2_) and superoxide (O_2_^−^) generation, can overwhelm glioblastoma antioxidant capacity and initiate oxidative cell death [13, 14, 15]. Hence, therapeutic strategies that target redox homeostasis have emerged as promising approaches for treatment‐refractory glioblastoma.

Ferroptosis is a novel, redox‐regulated, iron‐dependent form of cell death characterized by the accumulation of lethal lipid peroxides, offering a compelling strategy to eliminate glioblastoma cells [16, 17]. It bypasses the DNA repair‐mediated mechanisms that glioblastoma cells use to resist conventional treatments, providing an alternative mode of tumor cell death [18]. Elevated ROS levels activate ferroptosis in glioblastoma stem cells, which contribute to tumor recurrence [19]. It exploits GBM's metabolic vulnerabilities by increasing oxidative stress and iron accumulation, pushing cancer cells beyond their oxidative threshold and triggering cell death while sparing normal brain cells [20, 21]. Despite these advances, most pro‐oxidant therapies lack sufficient tumor selectivity, leading to systemic toxicity or adaptive antioxidant rebound responses that ultimately restore tumor survival. An ideal redox‐targeted therapy would therefore selectively amplify oxidative stress in glioblastoma cells without impairing antioxidant defenses or compromising immune function or normal tissue integrity [13, 22, 23].

*Rosasina* is a sodium chlorite (NaClO_2_)‐derived, redox‐active small molecule with high aqueous solubility [24] and controlled oxidative potential [25], properties that overcome key limitations of conventional pro‐oxidant therapies, including poor stability and limited tumor selectivity [26]. In biological systems, NaClO_2_ reacts with gastric hydrochloric acid to generate chlorine dioxide (ClO_2_) [27]. ClO_2_ is rapidly reduced to chlorite (ClO_2_^−^), the principal bioactive species responsible for its biological activity [28]. Pharmacokinetic studies have demonstrated that ClO_2_^−^ is efficiently absorbed following oral administration, widely distributed to peripheral tissues, including the brain, and rapidly eliminated through renal clearance, supporting systemic exposure while minimizing prolonged accumulation [24, 28]. Consistent with these pharmacological properties, ClO_2_^−^ has demonstrated favorable safety profiles in preclinical toxicology studies (EPA/635/R‐00/007) and early‐phase clinical trials (NCT03602066), with no significant treatment‐related toxicity observed at therapeutically relevant doses [27]. Mechanistically, chlorite participates in controlled redox reactions with hemoproteins [29, 30], generating secondary oxidants [30] and modulating oxidative stress [31], inflammatory signaling and immune responses [32, 33]. These pharmacological properties provide a strong rationale for investigating *Rosasina* as a redox‐targeted therapeutic that selectively exploits the heightened redox vulnerability of glioblastoma cells to induce ferroptosis while simultaneously remodeling the tumor immune microenvironment.

In this study, we investigated *Rosasina* as a redox‐targeted therapeutic that selectively exploits the redox vulnerabilities of glioblastoma cells to induce ferroptosis. We demonstrate that *Rosasina* promotes ROS accumulation and lipid peroxidation, leading to ferroptotic cell death. In an orthotopic glioblastoma model, *Rosasina* suppressed tumor growth, prolonged survival, and remodeled the tumor immune microenvironment through pro‐inflammatory M1‐like macrophage polarization. Together, these findings establish *Rosasina* as a novel mechanistically distinct therapeutic strategy that integrates redox modulation, ferroptosis induction, and immune remodeling, providing a strong rationale for its further preclinical and clinical development for the glioblastoma treatment.

## 2 Results

### 2.1 *Rosasina* Induces ROS Production in Glioblastoma Cells

To evaluate the ROS‐inducing activity of *Rosasina*, normal human astrocytes (NHA) and LN340 glioblastoma cells were treated with increasing concentrations of *Rosasina* (1–1000 nM) for up to 240 min. Intracellular ROS levels were quantified using dichloro‐dihydro‐fluorescein diacetate (DCF‐DA), which is oxidized by ROS to generate a fluorescent signal. Fluorescence microscopy revealed a clear concentration‐ and time‐dependent increase in DCF‐DA fluorescence in both cell types. Notably, LN340 cells exhibited substantially higher fluorescence intensity than NHA at equivalent concentrations and time points, indicating enhanced ROS production in glioblastoma cells following *Rosasina* treatment (Figure 1A). In contrast, NHA displayed minimal fluorescence, with detectable ROS induction observed only at the highest concentration (1000 nM) at early time points (30 min), consistent with limited ROS accumulation (Figure 1A, *upper panel*). Importantly, LN340 cells demonstrated robust ROS induction even at lower concentrations of *Rosasina* (10 nM) within 30 min of exposure, whereas NHA remained largely unresponsive. This differential response highlights the selective redox vulnerability of LN340 glioblastoma cells.

**FIGURE 1:**
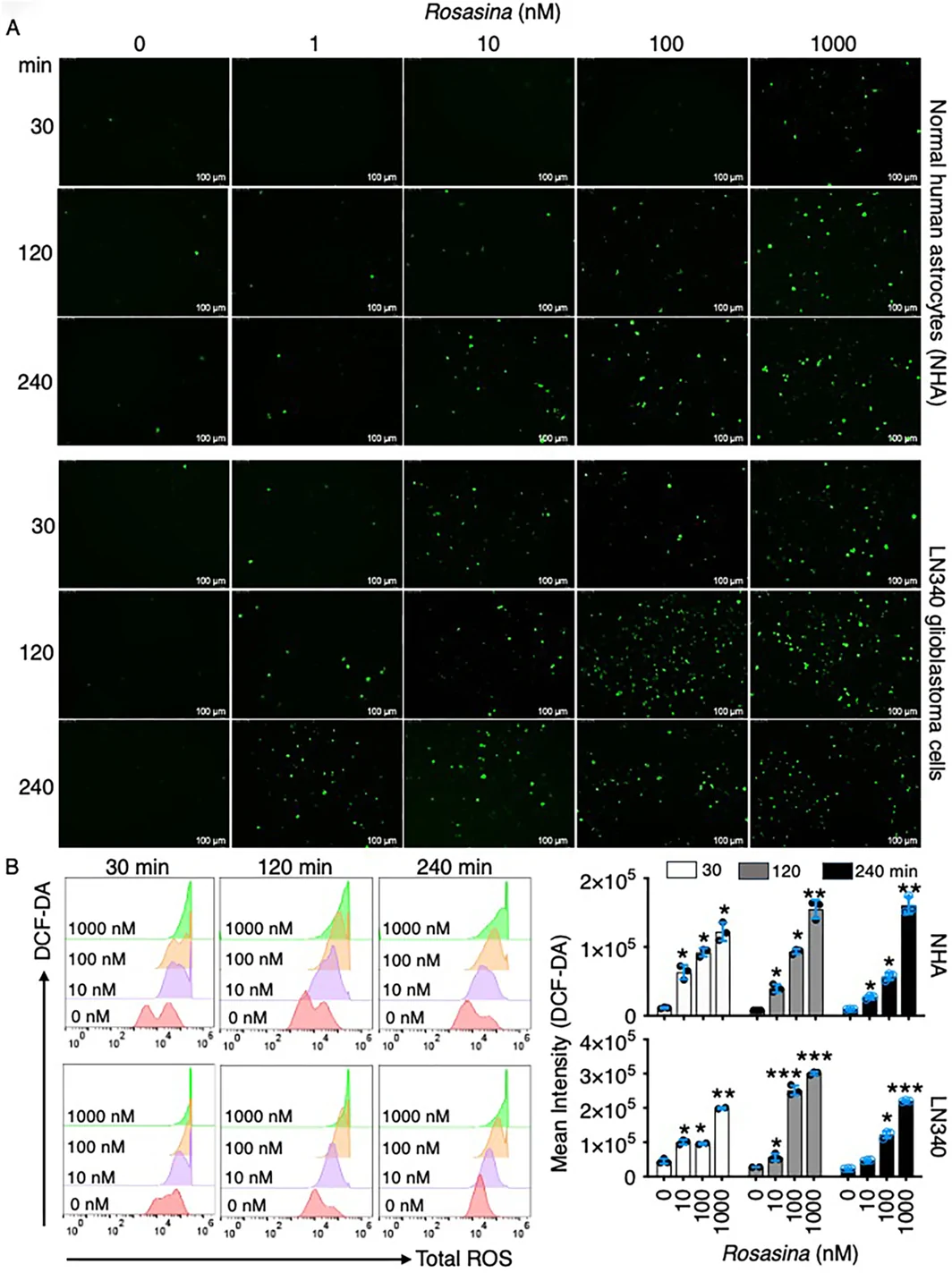
*Rosasina* induces dose‐dependent ROS generation. (A) Representative fluorescence microscopy images showing intracellular ROS generation in normal human astrocytes (NHA) and glioblastoma cells (LN340) following treatment with increasing concentrations of *Rosasina* (1, 10, 100, and 1000 nM) for 30, 120, and 240 min. Cells were incubated with the ROS‐sensitive fluorescent dye 2′,7′‐dichlorofluorescein diacetate (DCF‐DA) to detect intracellular ROS. Enhanced green fluorescence intensity indicates elevated ROS production. (B) Quantitative analysis of mean fluorescence intensity (MFI) of DCF‐DA using flow cytometry (FITC channel) in LN340 and NHA cells treated with indicated concentration *Rosasina*. MFI was measured after 30, 120, and 240 min of treatment across concentrations. *p ≤ 0.05, **p ≤ 0.01 and ***p ≤ 0.001.

Flow cytometric quantification of total ROS further corroborated the microscopic observations. DCF‐DA based ROS analysis demonstrated a clear time‐ and concentration‐dependent increase in ROS levels (Figure 1B, *left panel*). Consistently, mean fluorescence intensity (MFI) measurements revealed a progressive elevation in ROS accumulation in LN340 cells, reaching a maximum at 1000 nM after 120 min of treatment (Figure 1B, *right panel*). Notably, even at lower concentrations (10 nM), a moderate yet statistically significant (∼3‐fold) increase in ROS was detected as early as 30 min (Figure 1B, *right panel*). In contrast, NHA cells exhibited comparatively lower ROS accumulation, with only a modest increase in MFI observed at 1000 nm following prolonged exposure, indicating reduced susceptibility to ROS induction relative to LN340 cells (Figure 1B). Collectively, these findings demonstrate that *Rosasina* preferentially induces intracellular ROS accumulation in LN340 cells compared with NHA, consistent with intrinsic differences in redox homeostasis between malignant and non‐malignant cells.

### 2.2 *Rosasina* Preferentially Increases Hydrogen Peroxide (H_2_O_2_) Production to Elevate Intracellular ROS

Cancer cells sustain elevated intracellular ROS levels through the selective generation of specific reactive species that support metabolic reprogramming and proliferative signaling [2, 34, 35]. Building on our observation that *Rosasina* amplifies ROS production in LN340 cells compared with NHA, we next sought to define the specific reactive species contributing to ROS accumulation following *Rosasina* treatment. Intracellular levels of O_2_^−^ and H_2_O_2_ were quantified by flow cytometric analysis in both NHA and LN340 cells. *Rosasina* treatment did not significantly alter intracellular O_2_^−^ levels in either cell type across all examined concentrations (10, 100, 1000 nM) and time points (30, 120 and 240 min) (Figure 2A). The geometric mean fluorescence intensity (geo‐MFI) of O_2_^−^ remained comparable to untreated controls, indicating that *Rosasina* does not induce measurable O_2_^−^ accumulation (Figure 2A, *right panel*).

**FIGURE 2:**
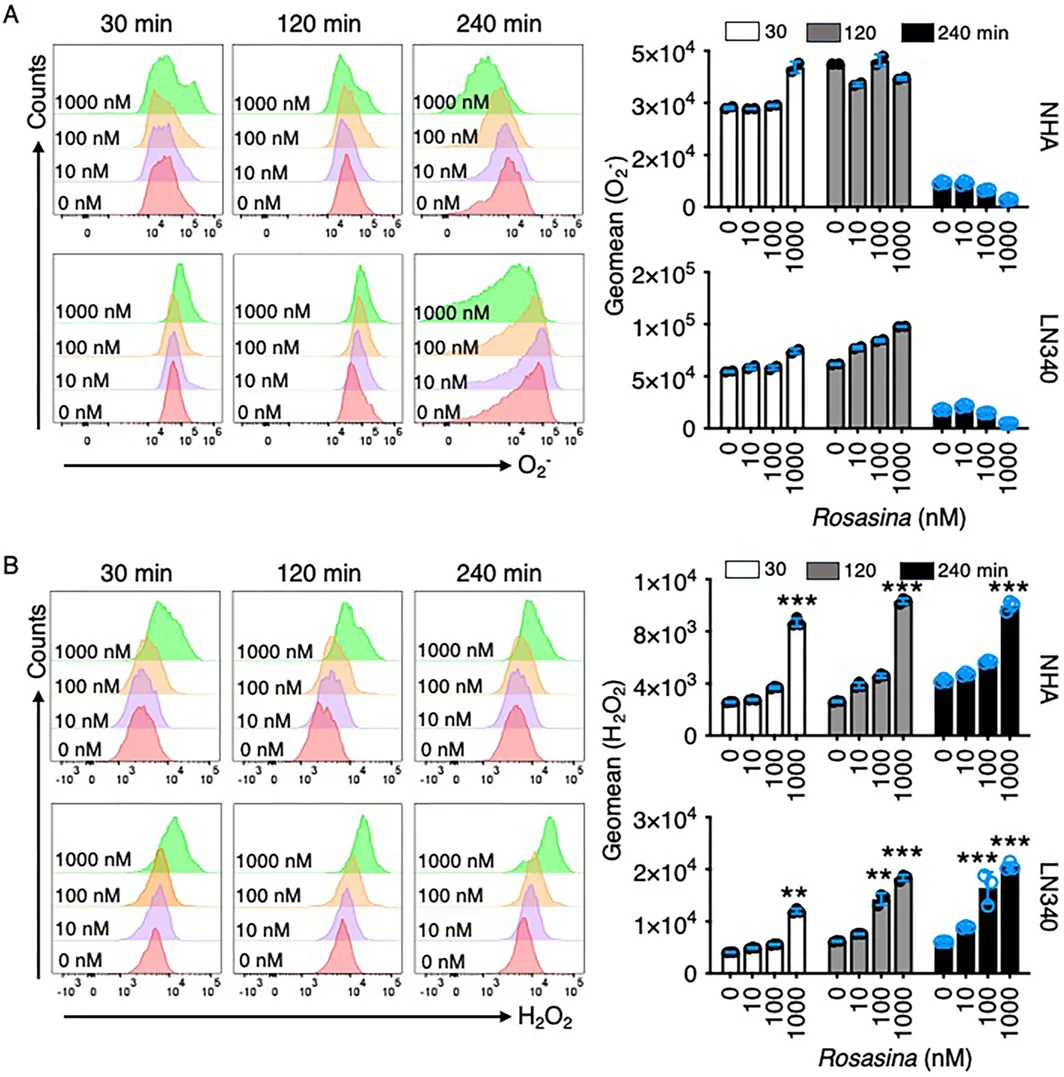
*Rosasina* induces hydrogen peroxide (H_2_O_2_) radical formation. (A) Normal human astrocytes (NHA) and LN340 glioblastoma cells were treated with increasing concentrations of *Rosasina* (10, 100, and 1000 nM) for 30, 120, and 240 min. Superoxide radical (O_2_^−^) generation was quantified using a flow cytometer equipped with the PE channel. The mean fluorescence intensity (MFI) of *Rosasina* ‐treated samples was compared with untreated control cells. (B) Hydrogen peroxide (H_2_O_2_) production was assessed in LN340 and NHA cells treated with *Rosasina* (10, 100, and 1000) for 30, 120, and 240 min. H_2_O_2_ generation was measured using a flow cytometer in the Pacific Blue channel. **p ≤ 0.01 and ***p ≤ 0.001.

In contrast, *Rosasina* markedly increased intracellular H_2_O_2_ levels in a concentration‐dependent manner in both cell types. In NHA, treatment with 1000 nM *Rosasina* resulted in a significant and sustained elevation of H_2_O_2_ at all evaluated time points (30, 120, and 240 min), indicating an oxidative response at higher concentrations (Figure 2B). Notably, LN340 cells exhibited a substantially greater and sustained increase in H_2_O_2_ production than NHA, as indicated by a broader fluorescence distribution and higher geo‐MFI, with significant peroxide accumulation detected at lower concentrations (≥ 100 nM) beginning at 120 min and persisting through 240 min (Figure 2B, *right panel*). Collectively, these results demonstrate that *Rosasina* selectively promotes H_2_O_2_ accumulation rather than O_2_^−^ generation, with a markedly enhanced effect in LN340 cells.

### 2.3 *Rosasina* Induces Transient ROS Accumulation in Peripheral Immune Cell Subsets

Given the established role of oxidative signaling in immune cell activation and function, we next assessed whether *Rosasina* modulates intracellular ROS levels in peripheral immune populations. Human peripheral blood mononuclear cells (PBMCs) were treated with increasing concentrations of *Rosasina* (10–1000 nM) for 30‐ or 120‐min. ROS levels were quantified by DCF‐DA‐based flow cytometry in CD3^+^ T cells, CD19^+^ B cells, CD14^+^ monocytes, and CD11c^+^ dendritic cells (DCs) (Supporting Information S1: Figure S1). *Rosasina* induced dose‐ and time‐dependent ROS accumulation across immune cell subsets, with distinct cell type‐specific magnitudes and kinetics. In CD3^+^ T cells, ROS levels increased significantly at 100 nm, most prominently at 30 min relative to untreated controls (Supporting Information S1: Figure S1A). This response was attenuated by 120 min, consistent with a transient oxidative burst. Treatment with 1000 nM resulted in variable ROS induction, characterized by modest early increases followed by a decline at later time points.

In CD19^+^ B cells, *Rosasina* increased ROS levels at both 10 and 100 nM, with maximal induction observed at 100 nM (Supporting Information S1: Figure S1B). Elevated ROS was detectable at 30 min and persisted, albeit at reduced levels, at 120 min. In contrast, CD14^+^ monocytes exhibited the most robust oxidative response (Supporting Information S1: Figure S1C). Treatment with 100 nM *Rosasina* induced a rapid and sustained increase in ROS that remained elevated through 120 min, whereas 1000 nM produced a pronounced early increase followed by partial normalization at later time points, indicating heightened sensitivity of this population to *Rosasina* ‐induced oxidative stress. CD11c^+^ dendritic cells displayed a more transient and non‐linear response (Supporting Information S1: Figure S1D). A sharp increase in ROS was observed at 100 nM after 30 min, followed by a rapid decline by 120 min. At 1000 nm, only modest ROS induction was detected, suggesting differential redox regulation compared with other immune subsets.

### 2.4 *Rosasina* Selectively Reduces Catalase Activity in Glioblastoma Cells

To assess whether *Rosasina* modulates endogenous antioxidant defenses contributing to intracellular ROS accumulation, we measured catalase (CAT) and superoxide dismutase (SOD) activities in NHA, LN340 glioblastoma cells, and PBMCs (Supporting Information S1: Figure S2). Cells were treated with increasing concentrations of *Rosasina* (10, 100, and 1000 nM) for 30, 120, and 240 min, and antioxidant enzyme activities were quantified using spectrophotometric assays. *Rosasina* did not significantly alter catalase (CAT) or superoxide dismutase (SOD) activity in NHA or PBMCs under any condition (Supporting Information S1: Figure S2A,B,E,F). In contrast, LN340 glioblastoma cells exhibited a pronounced time‐ and dose‐dependent reduction in CAT activity, with significant suppression observed at 1000 nM as early as 30 min and sustained inhibition at both 100 and 1000 nM at later time points (Supporting Information S1: Figure S2C). Notably, SOD activity remained unchanged across all treatment conditions (Supporting Information S1: Figure S2D). Consistent with these enzymatic changes, RT‐qPCR analysis revealed a ∼2‐fold decrease in CAT mRNA and a concomitant ∼2‐fold increase in SOD expression in LN340 cells (Supporting Information S1: Figure S3A,B). Similar expression pattern was observed by immunoblot analysis using specific antibodies (Supporting Information S1: Figure S3C,D), suggesting a compensatory response to oxidative stress. Collectively, these findings indicate that *Rosasina* selectively disrupts catalase‐mediated peroxide detoxification in glioblastoma cells, promoting sustained H_2_O_2_ accumulation without altering upstream superoxide dismutation, thereby reinforcing a tumor‐specific oxidative vulnerability.

### 2.5 In Vitro and In Vivo Safety Profile of *Rosasina*

To determine whether *Rosasina*'s redox activity translates into selective cytotoxicity to glioblastoma cells, we first evaluated its selective effects on NHA, LN340, LN229, MGG123, GBM10 and 12 cell lines using concentration‐dependent viability assays. Treatment of NHA cells with *Rosasina* (1–1000 nM) for 24 h resulted in minimal cytotoxicity, with > 85% cell viability maintained at 1000 nM and ≥ 99% viability observed at concentrations of 100 nM or lower (Figure 3A). Notably, NHA viability following *Rosasina* treatment was comparable to, or higher than, that observed with doxorubicin (Dox) treatment used as a control. These data indicate that non‐malignant astrocytes tolerate *Rosasina* exposure across a broad concentration range. In contrast, glioblastoma cells (LN340, LN229, MGG123, GBM10 and 12) displayed a concentration‐dependent reduction in viability following *Rosasina* treatment. A significant decrease in cell survival was evident beginning at 10 nm, with maximal cytotoxic effects observed at 1000 nM (Figure 3B). This differential response highlights a selective vulnerability of malignant cells to *Rosasina* exposure likely through mechanisms involving oxidative stress.

**FIGURE 3:**
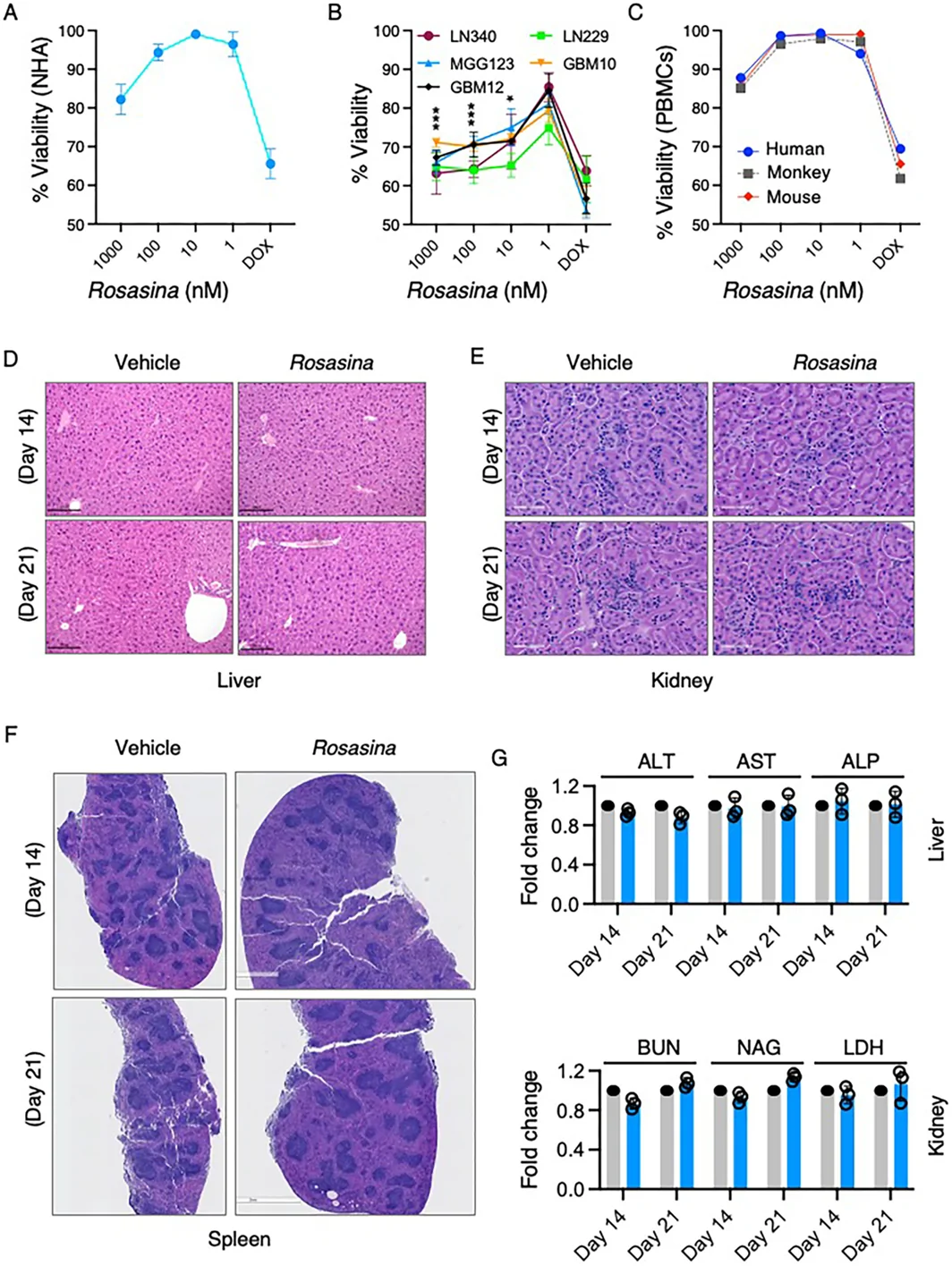
Cell viability and toxicity assessment of *Rosasina*. (A) Dose‐dependent cell viability analysis of normal human astrocytes (NHA) treated with increasing concentrations of *Rosasina* (1–1000 nM) for 24 h. Cell viability was determined using a standard MTT assay. Doxorubicin (Dox: 50 μm) used as control. (B) Viability assessment of various glioblastoma cell lines (LN340, LN229, MGG123, GBM10 and 12) treated with *Rosasina* (1–1000 nM) for 24 h. (C) Comparative cytotoxicity evaluation in peripheral blood mononuclear cells (PBMCs) derived from human, monkey, and mouse following exposure to various concentrations of *Rosasina* (1–1000 nM) for 24 h. (D–F) In vivo toxicity assessment of *Rosasina* in mice. C57BL/6J (*n* = 6: female‐3 and male‐3) mice were treated with *Rosasina* (2 mg/kg/day) or H_2_O as vehicle for 14 or 21 days. Subsequently, liver, kidney, and spleen tissues were collected for histopathological evaluation. Hematoxylin and eosin (H&E) staining of liver (D), kidney (E), and spleen (F). Representative images are shown. (G) Serum biochemical parameters evaluation for hepatotoxicity and nephrotoxicity following *Rosasina* treatment. Bar graph represents liver function markers such as alanine aminotransferase (ALT), aspartate aminotransferase (AST) and alkaline phosphatase (ALP), as well as kidney function markers, including blood urea nitrogen (BUN) and NAG (N‐acetyl‐β‐D‐glucosaminidase), in vehicle‐ and *Rosasina* ‐treated animals. Data are presented as mean ± SD. Statistical analysis was performed relative to vehicle‐treated controls.

Next, we examined whether *Rosasina* exerts a cytotoxic effect on non‐malignant primary immune cells across species. PBMCs isolated from human, non‐human primate, and mouse were treated with increasing concentrations of *Rosasina* for 24 h. MTT viability assays revealed that PBMC survival remained largely unchanged across all concentrations tested, with > 90% cell viability observed in each species (Figure 3C). These findings indicate that *Rosasina* exhibits selective cytotoxicity toward malignant cells while sparing non‐malignant cells, including circulating immune cell populations.

In vivo safety was further evaluated by histopathological and biochemical analyses of major organs from mice treated with *Rosasina* (2 mg/kg/day) once daily for up to 21 days (*n* = 6). Body weight was recorded throughout the treatment period. Organs were harvested on days 14 and 21, and organ weights were measured to assess potential treatment‐related abnormalities. No significant differences in body and organ weights were observed between *Rosasina* and vehicle‐treated mice (Supporting Information S1: Figure S4A,B and Table S1). Histological examination of hematoxylin and eosin (H&E)‐stained sections of liver (Figure 3D), kidney (Figure 3E) and spleen (Figure 3F) demonstrated preserved tissue architecture with no evidence of treatment‐related necrosis, inflammatory infiltration, or structural abnormalities in *Rosasina*‐treated mice compared with vehicle‐treated controls (Figure 3D–F). To further determine whether the histological changes occurred due to treatment‐related toxicity or spontaneous background lesions, blinded semi‐quantitative histopathological scoring was performed. The analysis identified only minimal background lesions, including focal to multifocal mixed inflammatory infiltrates, rare hepatocyte necrosis, extramedullary hematopoiesis, and non‐lipid hepatocellular vacuolar change (Supporting Information S1: Figure S5A–C). Importantly, the incidence and severity of these findings were comparable between vehicle‐ and *Rosasina*‐treated groups, indicating that they represented spontaneous background changes rather than treatment‐related toxicity.

Consistent with these findings, serum biochemical markers of liver (ALT, AST and ALP) and kidney (BUN, NAG and LDH) function were comparable between *Rosasina* and vehicle‐treated groups (Figure 3G). Together, these data indicate that *Rosasina* administered at 2 mg/kg/day is well tolerated in vivo under the conditions tested. Collectively, the in vitro and in vivo safety assessments demonstrate that *Rosasina* exhibits minimal off‐target toxicity, supporting its favorable safety profile as a therapeutic candidate for glioblastoma.

### 2.6 *Rosasina* Suppressed Glioblastoma Growth In Vivo

Having established the safety of *Rosasina*, we next evaluated its therapeutic efficacy in an orthotopic glioblastoma model. Luciferase‐expressing murine glioblastoma GL261 cells were stereotactically implanted into the brains of C57BL/6J mice, and tumor engraftment was confirmed by bioluminescence imaging seven days after implantation. Mice were then randomized to receive either *Rosasina* (2 mg/kg/day) or vehicle by oral gavage once daily until the experimental endpoint. Tumor progression was monitored by serial bioluminescence imaging for up to 21 days following treatment initiation, and animals were observed until survival endpoints (42 days).

Baseline bioluminescence intensities were comparable between treatment groups prior to therapy. In vehicle‐treated mice, bioluminescence increased progressively over time, reflecting continued tumor growth, whereas *Rosasina*‐treated mice exhibited markedly reduced photon flux throughout the observation period (*n* = 8 for *Rosasina* and vehicle treated groups, Figure 4A). Quantitative analysis demonstrated significantly lower tumor‐associated bioluminescence in *Rosasina*‐treated mice at both 14 days (*p* < 0.01) and 21 days (*p* < 0.0001) after treatment initiation (Figure 4B). Consistent with the reduced tumor burden, *Rosasina* treatment significantly prolonged survival, increasing the median survival from 27 days in vehicle‐treated mice to 35 days in *Rosasina* treated animals (*p* < 0.01; Figure 4C). These findings demonstrate that *Rosasina* effectively suppresses glioblastoma progression and significantly improves survival in vivo.

**FIGURE 4:**
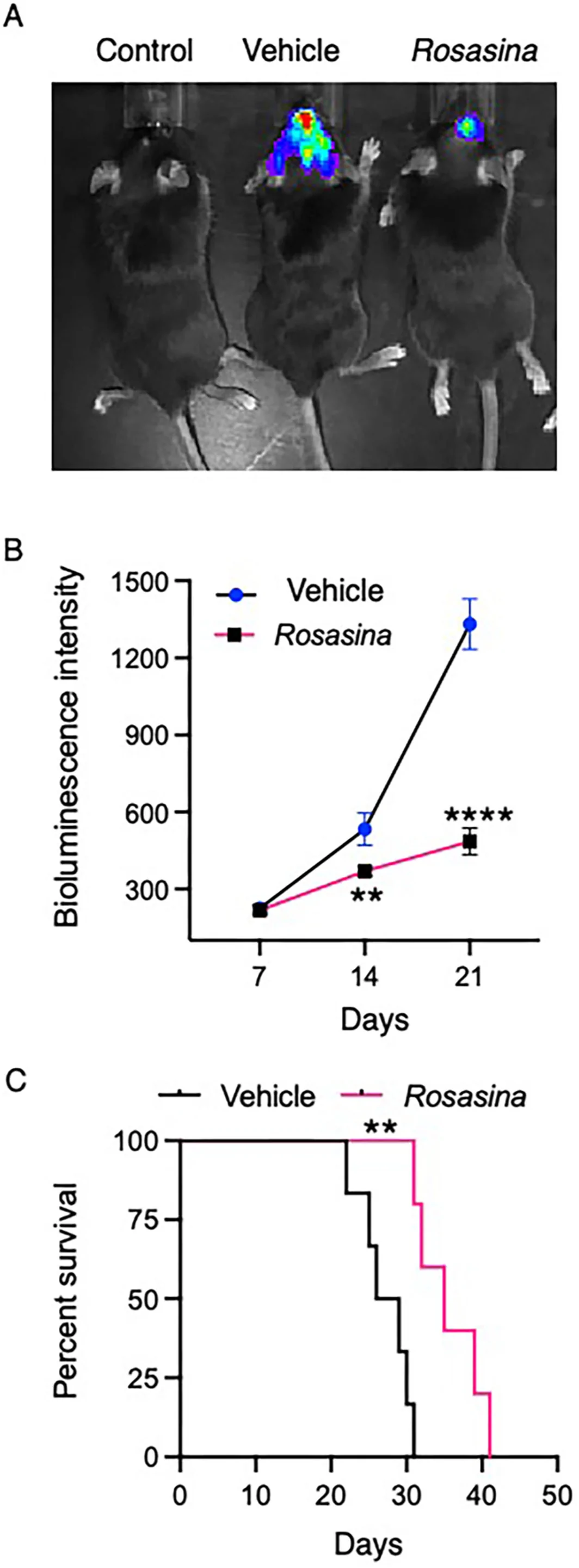
*Rosasina* inhibits glioblastoma growth and prolongs survival (A) Representative bioluminescence image of C57BL/6J mice bearing intracranial luciferase‐expressing GL261 tumors treated with vehicle or *Rosasina*. (B) Quantification of tumor bioluminescence at days 7, 14, and 21 after treatment initiation. Data are presented as mean ± SD. **p < 0.01; ***p < 0.001. (C) Kaplan Meier survival curves of *Rosasina*‐ and vehicle‐treated mice (**p < 0.01).

To determine whether prolonged *Rosasina* treatment induced systemic toxicity in tumor‐bearing animals, liver, kidney, and spleen tissue samples collected at the study endpoint were subjected to histopathological evaluation. H&E staining revealed preserved tissue architecture without evidence of treatment‐related structural abnormalities in *Rosasina* ‐treated mice compared with vehicle controls (Supporting Information S1: Figure S6A–C). Semi‐quantitative histopathological scoring identified only minimal background lesions that occurred at comparable frequency and severity in both treatment groups, consistent with the findings observed in healthy *Rosasina*‐treated mice (Figure 3D–F and Supporting Information S1: Figure S5). These results indicate that the antitumor efficacy of *Rosasina* is achieved without detectable systemic toxicity in tumor‐bearing mice.

### 2.7 *Rosasina* Promotes Ferroptosis‐Mediated Glioblastoma Cell Death

To understand the mechanism by which *Rosasina* reduce tumor growth and determine whether the reduction in glioblastoma cell viability is mediated by programmed cell death, we assessed apoptosis using Annexin V/propidium iodide (PI) staining and flow cytometry. NHA and LN340 glioblastoma cells were treated with increasing concentrations of *Rosasina* and analyzed across multiple time points. At concentrations ranging from 1 to 100 nM, *Rosasina* did not induce a significant increase in Annexin V‐positive cells in either NHA or LN340 cells compared with untreated controls (0 nM) (Supporting Information S1: Figure S7, *left to right*). At a higher concentration (1000 nM) and extended exposure (240 min), a modest increase in late apoptotic (Annexin V^+^/PI^+^) cell populations was observed, accompanied by a relative decrease in early apoptotic (Annexin V^+^/PI^−^) cells compared with untreated controls (0 nM) (Supporting Information S1: Figure S7, *right bottom panel*). However, this effect was comparable between NHA and LN340 cells and did not show a tumor‐selective increase in apoptosis. Collectively, these findings indicate that *Rosasina* ‐induced decrease in glioblastoma cell viability is not primarily mediated by apoptosis but rather occurs through a non‐apoptotic cell death mechanism.

Given that *Rosasina* ‐induced reduction in glioblastoma cell viability was not mediated by apoptosis, we next investigated whether an alternative programmed cell death pathway ferroptosis was involved. To test this possibility, we assessed oxidative lipid damage and intracellular iron accumulation in LN340 cells, with NHA serving as a non‐malignant control (Figure 5A). Lipid peroxidation was evaluated using the fluorescent lipid sensor BODIPY‐C11, which undergoes a shift in emission from red (reduced) to green (oxidized) upon lipid oxidation [36]. Under basal conditions, LN340 cells predominantly exhibited red fluorescence, indicating low basal lipid peroxidation. Following 24 h of *Rosasina* treatment, LN340 cells displayed a marked increase in green fluorescence, consistent with substantial lipid peroxide accumulation (Figure 5A, *right panel*). In contrast, NHA showed only minimal changes in BODIPY‐C11 oxidation (Figure 5A, *left panel*). Merged images further confirmed the predominance of oxidized BODIPY‐C11 signal in *Rosasina* ‐treated LN340 cells. Similarly, co‐treatment with the ferroptosis inhibitor Ferrostatin‐1 significantly attenuated *Rosasina*‐induced lipid peroxidation in LN340 cells (Figure 5A, *right panel*), further supporting that *ROSASINA* induces oxidative lipid damage through a ferroptosis‐dependent mechanism. Erastin, a well‐established ferroptosis inducer, was included as a positive control.

**FIGURE 5:**
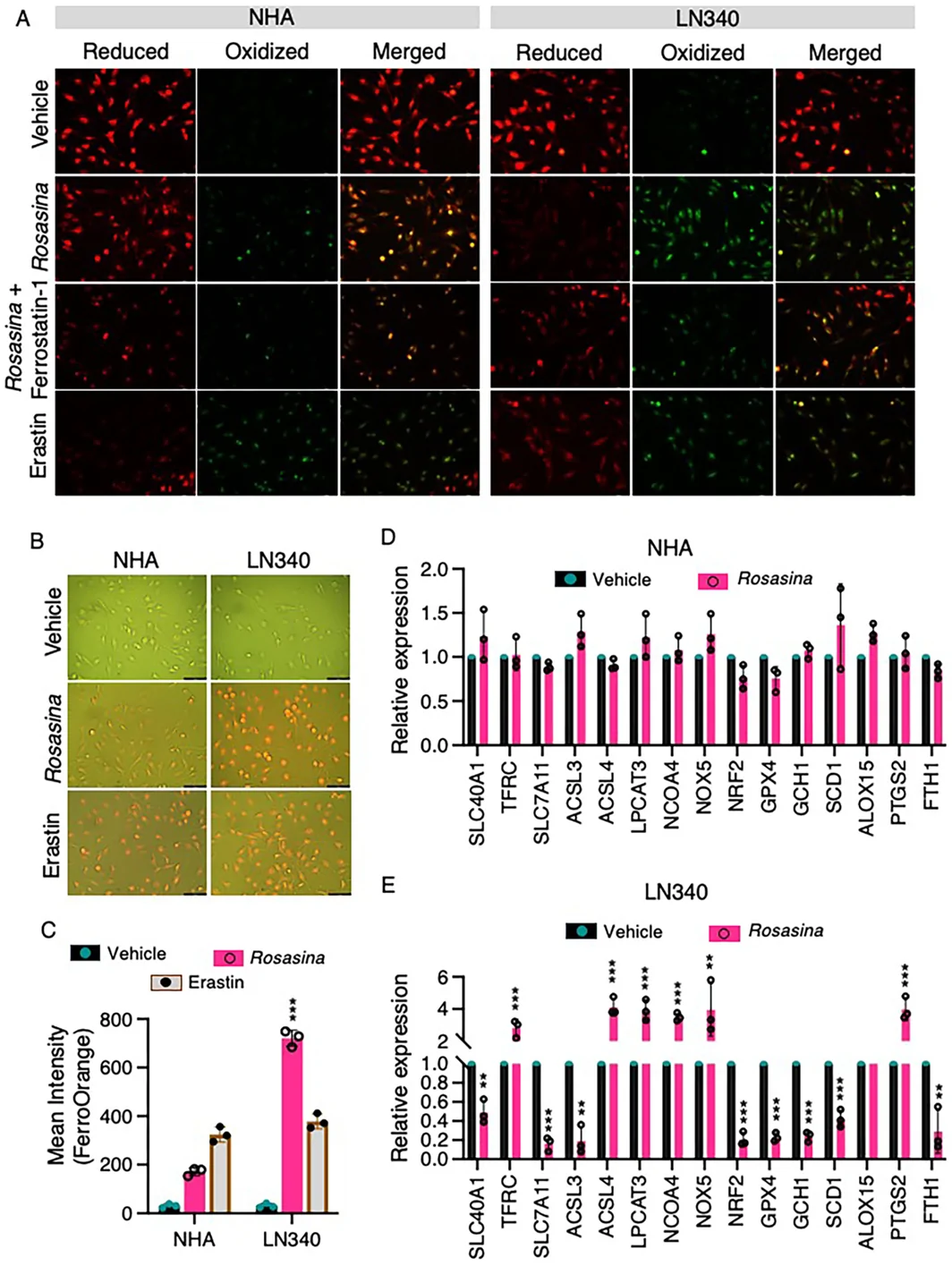
*Rosasina* induces ferroptosis‐associated lipid peroxidation and iron accumulation in glioblastoma cells. (A) Lipid peroxidation was assessed using BODIPY‐C11 staining in NHA and LN340 cells following 24 h *Rosasina* treatment. Representative fluorescence images show oxidized (red) and reduced (green) BODIPY signals, with merged images presented in the right panels. Ferrostatin‐1, a ferroptosis inhibitor, and Erastin, a ferroptosis inducer, were included as negative and positive controls, respectively. Representative images were processed using identical brightness and contrast settings across all experimental groups to enable direct visual comparison. (B) Intracellular ferrous iron (Fe^2+^) accumulation was assessed using FerroOrange staining. Representative fluorescent images demonstrate iron accumulation. (C) Quantification of FerroOrange mean fluorescence intensity of intracellular iron levels in *Rosasina*‐treated LN340 cells relative to untreated controls and NHA cells. (D–E) Quantitative reverse transcriptase PCR (RT‐qPCR) analysis of ferroptosis‐related genes in NHA (D) and LN340 (E) cells after *Rosasina* (10 nM) treatment. ***p < 0.001 between untreated and *Rosasina* treated groups (Student's *t*‐test).

To determine whether lipid peroxidation was accompanied by altered iron homeostasis, a hallmark of ferroptosis. We next measured intracellular ferrous iron (Fe^2+^) levels using FerroOrange staining (Figure 5B). *Rosasina* ‐treated LN340 cells exhibited a marked increase in FerroOrange fluorescence, indicating expansion of the labile iron pool (Figure 5B, *right panel*). In contrast, NHA displayed minimal changes in FerroOrange intensity (Figure 5B, *left panel*). Quantitative analysis of mean fluorescence intensity confirmed a significant increase in intracellular Fe^2+^ levels in LN340 cells relative to untreated controls (Figure 5C). Together, these findings demonstrate that *Rosasina* induces coordinated lipid peroxidation and iron accumulation in glioblastoma cells, supporting ferroptosis as a key mechanism underlying *Rosasina*‐mediated cytotoxicity.

### 2.8 *Rosasina* Modulates Ferroptosis‐Related Gene Expression in Glioblastoma Cells

To further substantiate the involvement of ferroptosis, we examined the expression of key ferroptosis‐associated genes by RT‐qPCR (Figure 5D,E). In LN340 cells, *Rosasina* treatment significantly upregulated genes involved in iron uptake and lipid peroxidation, including *TFRC,* and *NCOA4*, exhibiting approximately 3‐fold induction relative to vehicle‐treated controls. Expression of *SLC11A2, ACSL4, LPCAT3, PTGS2* and *NOX5* was increased by approximately 4‐fold, respectively. In contrast, *Rosasina* treatment markedly suppressed the expression of genes associated with antioxidant defense and ferroptosis resistance, including *SLC40A1*, *SLC7A11, ACSL3, NRF2, GPX4, GCH1, SCD1* and *FTH1*, each reduced by approximately 5‐fold (Figure 5E).

By comparison, NHA exhibited non‐significant changes in the examined gene expression following *Rosasina* treatment relative to vehicle‐treated controls (Figure 5D), consistent with the limited lipid peroxidation and iron accumulation observed in these cells (Figure 5A–C). This differential transcriptional response supports a selective ferroptotic vulnerability in glioblastoma cells and is consistent with intrinsic differences in redox homeostasis, iron metabolism, and antioxidant capacity between malignant and non‐malignant cells [37].

### 2.9 *NCOA4* and *NOX5* as Key Effectors of *Rosasina*‐Induced Ferroptosis of Glioblastoma Cells

To identify the key mediators of *Rosasina* ‐induced ferroptosis, we prioritized *NCOA4* and *NOX5* based on their coordinated upregulation in the ferroptosis‐associated transcriptional program (Figure 5E). Efficient siRNA‐mediated silencing of *NCOA4* and *NOX5* was confirmed by western blot analysis (Supporting Information S1: Figure S8A). To determine whether either gene contributes to basal cell survival or *Rosasina*‐mediated cytotoxicity, cell viability was assessed in the presence and absence of *Rosasina*. Under untreated conditions, depletion of *NCOA4* or *NOX5* did not significantly affect cell viability compared with non‐targeting siRNA (*siNT*)‐transfected controls, indicating that neither gene is required for basal cell survival. In contrast, *Rosasina* markedly reduced the viability of *siNT*‐transfected cells, whereas silencing of either *NCOA4* or *NOX5* significantly (*p* < 0.001) attenuated its cytotoxic effects (Figure 6A), demonstrating that both genes are required for *Rosasina* ‐mediated cytotoxicity.

**FIGURE 6:**
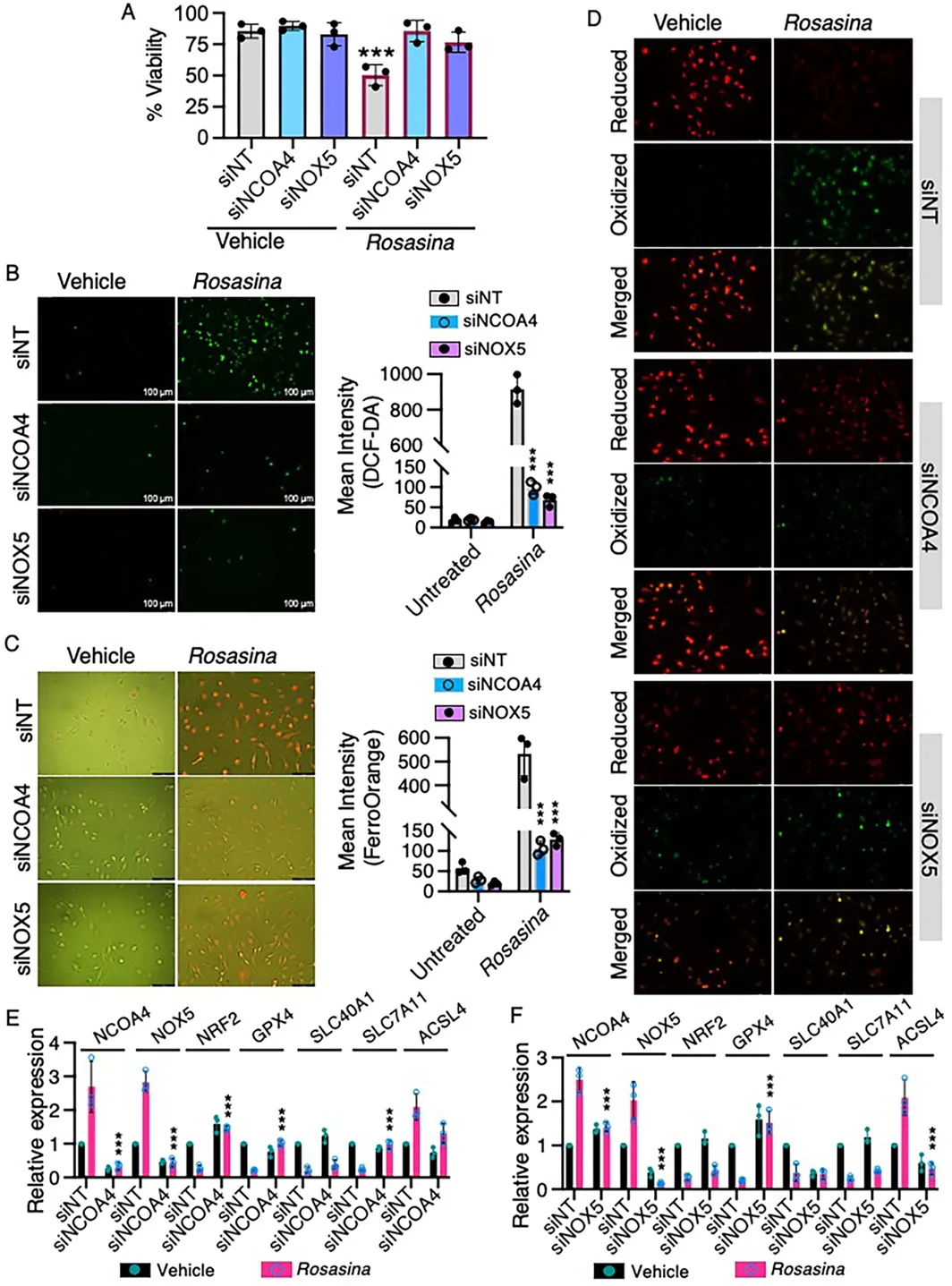
*NCOA4* and *NOX5* regulate ROS generation and lipid peroxidation in glioblastoma cells following *Rosasina* treatment. (A) Cell viability assessment of siRNA‐mediated knockdown of *NCOA4* and *NOX5* in LN340 cells treated with or without *Rosasina* (10 nM) for 24 h. Cell viability was determined using a standard MTT assay. (B) Representative fluorescence images showing DCF‐DA based detection of intracellular reactive oxygen species (ROS) following *Rosasina* treatment in control and knockdown cells. Quantification of mean fluorescence intensity (MFI) is shown on the right (mean ± SD). (C) FerroOrange‐based fluorescence detection of intracellular iron accumulation after *Rosasina* treatment in *siNT*, *siNCOA4* and s*iNOX5* transfected cells. Quantification of fluorescence intensity is shown on the right (mean ± SD). (D) Fluorescent detection of lipid peroxidation using the BODIPY‐C11 probe in *siNT*, *siNCOA4, and siNOX5* cells with or without *Rosasina*. Red and green fluorescence denote oxidized and reduced BODIPY forms, respectively; merged images depict oxidative status at the single image. (E, F) Quantitative RT‐PCR analysis of ferroptosis‐associated gene expression following *NCOA4* knockdown (E) and *NOX5* knockdown (F). ***p < 0.001 between untreated and *Rosasina* treated groups (Student's *t*‐test).

Consistent with their established biological functions, *NCOA4*, a ferritinophagy cargo receptor, promotes ferritin degradation and iron release, thereby expanding the labile iron pool and facilitating lipid peroxidation‐dependent ferroptosis [38, 39, 40]. In contrast, *NOX5* functions as a major enzymatic source of intracellular hydrogen peroxide, contributing to oxidative stress and ROS generation [41, 42, 43]. To determine whether either gene regulates basal oxidative stress, intracellular ROS levels were first measured in untreated cells. Baseline ROS production was comparable between non‐targeting control siRNA‐ and *NCOA4*‐or *NOX5*‐knockdown cells, indicating that depletion of either gene did not alter basal redox homeostasis in the absence of *Rosasina* (Figure 6B). In contrast, *Rosasina* treatment markedly increased intracellular ROS levels in control siRNA‐transfected cells, whereas this response was significantly attenuated following *NCOA4* or *NOX5* knockdown (Figure 6B). Consistent with these findings, siRNA‐mediated silencing of either *NCOA4* or *NOX5* significantly attenuated *Rosasina*‐induced expansion of the labile iron pool (Figure 6C) and lipid peroxidation (Figure 6D) compared with control siRNA‐transfected cells, demonstrating that both iron mobilization and ROS production are essential for *Rosasina*‐mediated ferroptotic signaling. Conversely, ectopic overexpression of *NCOA4* or *NOX5* (Supporting Information S1: Figure S8B) potentiated *Rosasina*‐induced cytotoxicity (Supporting Information S1: Figure S8C) and further enhanced lipid peroxidation (Supporting Information S1: Figure S8D). Together, these complementary loss‐ and gain‐of‐function studies establish *NCOA4* and *NOX5* as critical mediators of *Rosasina*‐induced ferroptosis by coordinating iron homeostasis, oxidative stress, and lipid peroxidation.

To further define the molecular mechanisms underlying *Rosasina*‐induced ferroptosis, we next examined whether *NCOA4* and *NOX5* regulate key antioxidant defense pathways. In control siRNA‐transfected cells, *Rosasina* induced an approximately threefold increase in *NCOA4* and *NOX5* expression while concomitantly suppressing the antioxidant regulators *NRF2, GPX4,* and *SLC7A11* (Figure 6E,F), consistent with activation of iron mobilization and oxidative stress signaling. Silencing *NCOA4* significantly restored the expression of *NRF2, GPX4*, and *SLC7A11* following *Rosasina* treatment, whereas *NOX5* knockdown increased *GPX4* expression and reduced *ACSL4* expression (Figure 6E,F). These findings demonstrate functional crosstalk between pro‐oxidant signaling and antioxidant defense pathways and identify *NCOA4* and *NOX5* as complementary mediators of *Rosasina*‐induced ferroptosis through coordinated regulation of iron homeostasis, ROS generation, lipid peroxidation, and antioxidant signaling.

### 2.10 *Rosasina* Promotes Accumulation of M1‐Like Microglia/Macrophages Within the Glioblastoma Microenvironment

Beyond its direct cytotoxic and antitumor effects, oxidative stress has been shown to regulate antitumor immune responses [44, 45]. Therefore, we next investigated whether *Rosasina* altered the immune composition of the glioblastoma tumor microenvironment. Mice bearing the orthotopic GFP‐labeled GL261 glioblastomas [46] were randomly assigned to receive either *Rosasina* or vehicle treatment for 21 days (*n* = 6), after which tumors were harvested for histological and immunological analyses. Consistent with the bioluminescence analysis (Figure 4A,B), H&E‐stained tumor sections demonstrated a marked reduction in tumor volume in *Rosasina*‐treated mice compared with vehicle‐treated controls (Figure 7A, *upper panel*). Immunohistochemical staining for Iba‐1 revealed a significant increase in tumor‐associated microglia/macrophages in *Rosasina*‐treated tumors compared with vehicle‐treated controls (*p* < 0.01; Figure 7A, *lower panel* and B), suggesting enhanced recruitment or expansion of myeloid cells within the tumor microenvironment.

**FIGURE 7:**
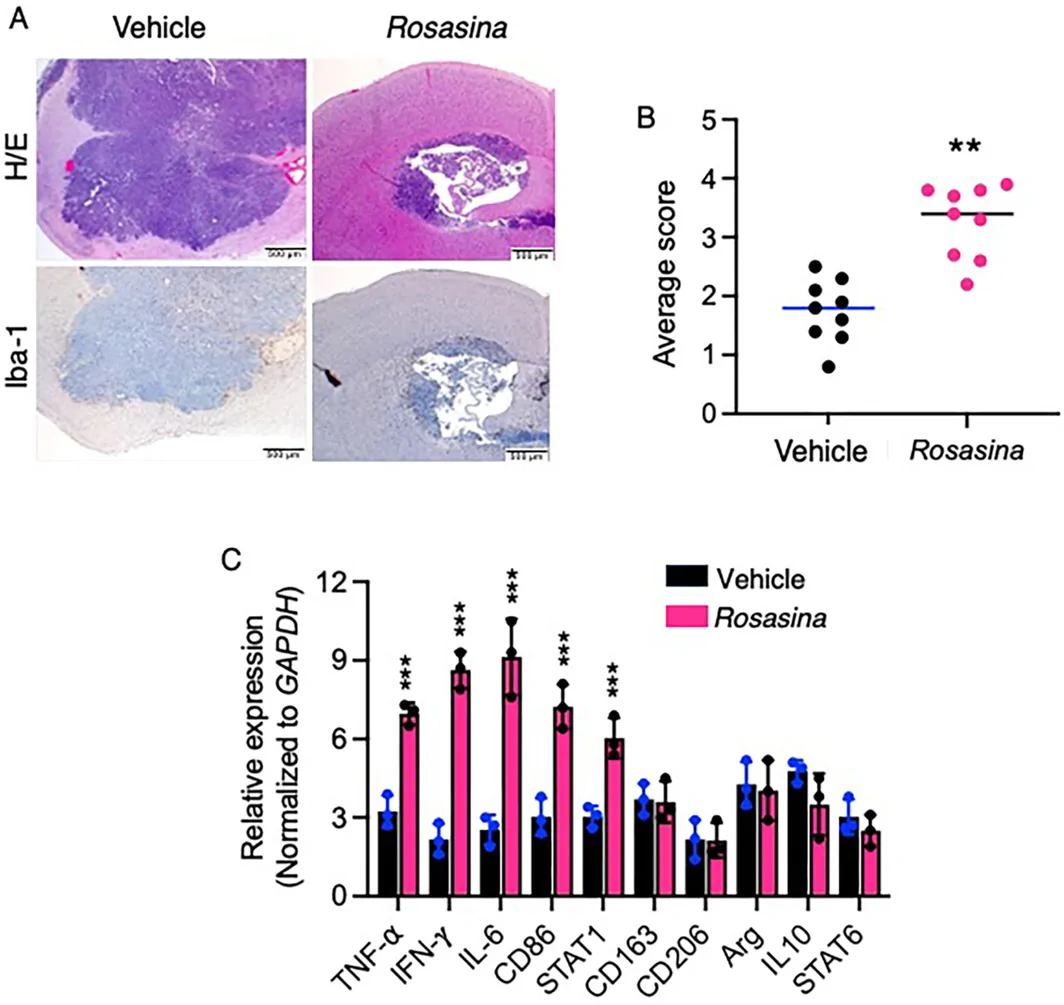
*Rosasina* promotes M1‐like microglia/macrophage polarization in the glioblastoma tumor microenvironment. (A) Representative hematoxylin and eosin (H&E) (top) and Iba‐1 immunohistochemical (bottom) staining of GFP‐GL261 tumors harvested from vehicle‐ or *Rosasina*‐treated mice after 21 days of treatment. *Rosasina*‐treated tumors exhibited markedly reduced tumor volume and increased Iba1‐positive microglia/macrophage infiltration compared with vehicle‐treated controls. (B) Quantification of Iba1‐positive microglia/macrophages. Ten randomly selected high‐power fields (400× magnification) were analyzed per tumor (5 intratumoral and 5 peritumoral fields). **p < 0.01. (C) Expression of indicated M1‐and M2‐associated genes in GFP^−^CD45^+^CD11b^+^ microglia/macrophage isolated from single‐cell suspensions of vehicle‐ and *Rosasina*‐treated GFP‐GL261 tumors. Gene expression was normalized to *GAPDH*. ***p < 0.001.

To determine whether these infiltrating myeloid cells acquired a pro‐inflammatory phenotype, GFP^−^CD45^+^CD11b^+^ microglia/macrophages were isolated from freshly resected GFP‐GL261 tumors by fluorescence‐activated cell sorting and the expression of polarization markers was analyzed by RT‐qPCR [46]. Compared with vehicle‐treated tumors, microglia/macrophages isolated from *Rosasina*‐treated mice exhibited robust upregulation of M1‐associated genes, including *TNF‐α* (∼2‐folds), *IFN‐γ* (∼3‐folds), *IL‐6* (∼3‐folds), *CD86* (∼3‐folds), and *STAT1* (∼2‐folds) (*p* < 0.001; Figure 7C). In contrast, the expression of M2‐associated markers (*CD163, CD206, ARG1, IL‐10*, and *STAT6*) did not exhibit significant changes. These findings demonstrate that *Rosasina* selectively promotes the accumulation of M1‐like microglia/macrophages without inducing an M2‐like immunosuppressive phenotype, suggesting that its antitumor activity extends beyond direct tumor cell killing to include remodeling of the glioblastoma immune microenvironment toward a pro‐inflammatory state.

### 2.11 *Rosasina* Remodels Macrophage Polarization Through Tumor‐Derived Paracrine Signaling

Emerging evidence suggests that ferroptotic tumor cells actively remodel the immune microenvironment through the release of redox‐dependent paracrine mediators [45, 47, 48]. We investigated whether the macrophage‐polarizing effects of *Rosasina* were driven by tumor‐derived signals generated during ferroptosis rather than by direct drug exposure. To address this question, LN340 glioblastoma cells were transfected with siRNAs targeting the key ferroptosis mediators *NCOA4* or *NOX5*, treated with *Rosasina*, and subsequently co‐cultured with primary human macrophages using an indirect Transwell system that selectively examined paracrine signaling (Figure 8A).

**FIGURE 8:**
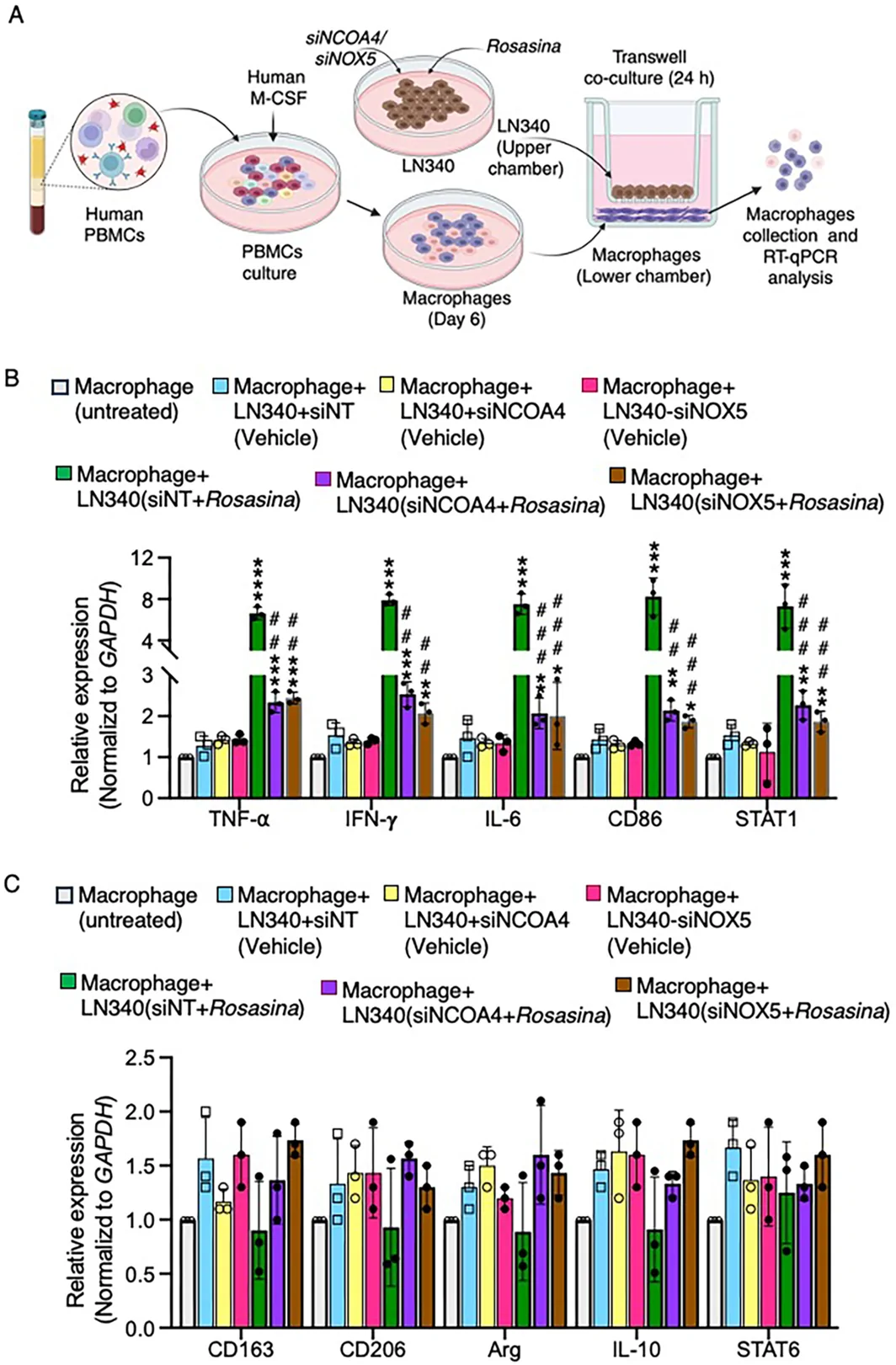
Inhibition of *NCOA4*‐ and *NOX5* disrupts *Rosasina*‐induced paracrine signaling and macrophage polarization. (A) Schematic illustration of the Transwell co‐culture of LN340 cells transfected with control (*siNT*), *siNCOA4*, or *siNOX5,* treated with or without *Rosasina* (10 nM) and co‐cultured with primary human macrophages to evaluate tumor cell‐mediated paracrine regulation of macrophage polarization. Macrophages were collected from the lower chamber of the Transwell, RNA was isolated and RT‐qPCR analysis of M1‐associated genes (*TNF‐α, IFN‐γ, IL‐6, CD86*, and *STAT1*) (B) and M2‐associated genes (*CD163, CD206, ARG1, IL‐10*, and *STAT6*) (C). Gene expression was normalized to GAPDH. Data are presented as mean ± SD. * and ^#^ *p* < 0.05; **and ^##^ *p* < 0.01; *** and ^###^ *p* < 0.001; ***p < 0.0001. *Compared to macrophage; #Compared to macrophage + LN340‐*siNT* + *Rosasina.*

Under basal conditions, macrophages co‐cultured with *NCOA4*‐or *NOX5*‐silenced LN340 cells in the absence of *Rosasina* exhibited no significant differences in the expression of the M1‐associated genes *TNF‐α, IFN‐γ, IL‐6, CD86, STAT1* (Figure 8B) or M2‐associated markers *CD163, CD206, ARG1, IL‐10*, and *STAT6* (Figure 8C), compared with macrophages co‐cultured with *siNT*‐transfected LN340 cells. These findings indicate that depletion of either *NCOA4* or *NOX5* alone does not substantially alter the basal secretory phenotype of glioblastoma cells or influence macrophage polarization.

In contrast, silencing either *NCOA4* or *NOX5* significantly attenuated the pro‐inflammatory macrophage response induced by *Rosasina*‐treated glioblastoma cells. Compared with macrophages co‐cultured with *Rosasina*‐treated *siNT*‐transfected LN340 cells, macrophages exposed to *Rosasina*‐treated *NCOA4*‐or *NOX5*‐deficient LN340 cells exhibited markedly reduced expression of *TNF‐α* (∼3‐fold), *IFN‐γ* (∼3.5‐fold), *IL‐6, CD86*, and *STAT1* (each ∼4‐fold; Figure 8B). In contrast, the expression of M2‐associated genes (*CD163, CD206, ARG1, IL‐10,* and *STAT6*) did not show a significant change in their expression (Figure 8C). These findings demonstrate that *NCOA4*‐and *NOX5*‐dependent ferroptotic signaling in glioblastoma cells is required for *Rosasina*‐induced activation of pro‐inflammatory M1‐like macrophages, while exerting minimal effects on M2‐associated polarization.

### 2.12 *Rosasina* Directly Reprograms Primary Human Macrophages Toward Pro‐Inflammatory M1‐Like Phenotype

To determine whether *Rosasina* directly reprogram macrophages toward a pro‐inflammatory M1‐like phenotype independent of tumor‐derived factors, human monocyte‐derived macrophages were differentiated in the presence of M‐CSF and treated with increasing concentrations of *Rosasina* (1, 10, and 100 nM). Flow‐cytometric analysis of canonical M1 and M2 surface markers revealed a concentration‐dependent increase in M1‐associated markers following *Rosasina* treatment (Figure 9A–F). Compared with untreated controls, *Rosasina* significantly increased *IFN‐γ* expression at 10 nM (32.2% ± 4.7%) and 100 nM (49.4% ± 5.9%) relative to control cells (20.16% ± 2.48%). Similarly, MHC‐II expression increased from 17.1% ± 1.2% in untreated macrophages to 30.3% ± 5.8% and 48.6% ± 9.7% at 10 and 100 nM, respectively, consistent with enhanced inflammatory activation and antigen‐presenting capacity (Figure 9C,F). In contrast, 1 nM *Rosasina* induced only modest changes (*IFN‐γ*: 24.43% ± 3.82%; MHC‐II: 22.37% ± 2.5%).

**FIGURE 9:**
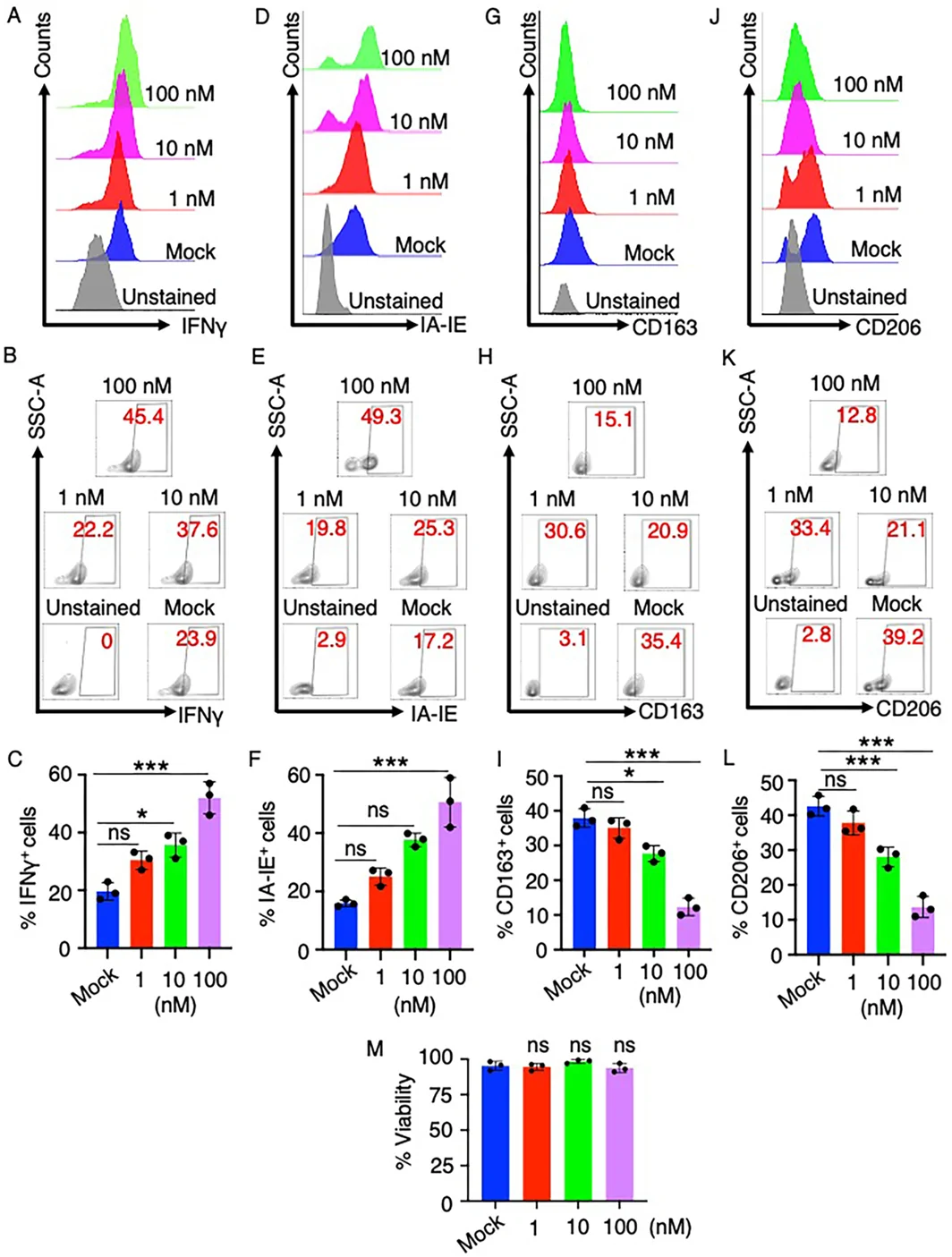
*Rosasina* drives the pro‐inflammatory polarization of primary human macrophages. Primary human monocyte‐derived macrophages were generated by differentiation in M‐CSF and subsequently treated with increasing concentrations of the ROS‐inducing agent *Rosasina* (1, 10, and 100 nM). Following treatment, macrophage polarization was assessed by multiparameter flow cytometry using established M1‐and M2‐associated surface markers. Cells were gated on live, single CD45^+^/CD14^+^ macrophages prior to analysis. (A–F) Representative overlaid histograms and contour plots showing expression of M1‐associated markers. *IFN‐γ* (A, B) and MHC‐II (D, E) across treatment conditions. Quantification of M1 marker expression, presented as percentage of positive cells (C, F). (G–L) Representative overlaid histograms and contour plots showing the expression of M2‐associated markers. CD206 (G, H) and CD163) (J, K). Quantification of M2 marker expression (I, L) across treatment groups. (M) Cell viability following *Rosasina* exposure assessed by MTT assay to evaluate potential cytotoxic effects of treatment. Data represent independent experiments performed with primary human donors (*n* = 4/group) in biological replicates. Quantified data are presented as mean ± SD. Statistical analysis was performed using ANOVA, with significance indicated as *p* < 0.05 compared with mock‐treated controls.

In parallel, *Rosasina* markedly suppressed the expression of M2‐associated markers (Figure 9G–L). Relative to mock‐treated cells (CD206: 36.8% ± 3.5%; CD163: 41.4% ± 2.5%), treatment with 10 nM *Rosasina* reduced CD206 and CD163 expression to 25.14% ± 3.8% and 24.92% ± 3.6%, respectively, whereas 100 nM treatment further decreased their expression to 14.0% ± 2.3% and 13.9% ± 1.6%. Minimal reductions were observed at 1 nM (CD206: 35.2% ± 5.7%; CD163: 37.0% ± 4.2%) (Figure 9I,L). To exclude the possibility that these effects resulted from cytotoxicity, macrophage viability was assessed by MTT assay. *Rosasina* did not significantly alter macrophage viability at any concentration tested compared with mock‐treated controls, indicating that the observed phenotypic changes reflect active immune reprogramming rather than loss of viable cells (Figure 9M).

Together, these findings demonstrate that *Rosasina* promotes a robust dose‐dependent shift toward an M1‐like macrophage phenotype, characterized by pro‐inflammatory and antigen‐presentation markers alongside suppression of M2‐associated markers. These results suggest that *Rosasina*‐mediated oxidative signaling can directly reprogram macrophages toward a pro‐inflammatory M1‐like phenotype and may contribute to anti‐tumor immune activation.

## 3 Discussion

Redox signaling pathways have emerged as central therapeutic targets across infectious, inflammatory, and malignant diseases, where oxidative imbalance critically governs cellular survival, immune function, and disease progression [49]. However, antioxidant‐based approaches have largely failed to yield effective cancer therapies because tumors adapt to intrinsically elevated ROS by reinforcing antioxidant defense systems [50, 51, 52]. These limitations have shifted the therapeutic focus toward pro‐oxidant strategies that overwhelm tumor antioxidant capacity and drive oxidative stress beyond cytotoxic thresholds.

In this study, we identified *Rosasina* as a selective ferroptosis inducer in glioblastoma that operates through coordinated disruption of redox homeostasis and inhibition of the SLC7A11‐*GPX4* antioxidant axis [45, 53]. *Rosasina* induced a pronounced accumulation of H_2_O_2_ in LN340 cells together with reduced catalase activity, establishing a sustained oxidative burden that exceeds the antioxidant capacity of glioblastoma cells. Notably, H_2_O_2_ accumulation occurred in the absence of detectable increase in O_2_^−^ or changes in SOD activity, suggesting that SOD is unlikely to be the primary source of H_2_O_2_. Although the mechanism responsible for this selective H_2_O_2_ accumulation was not investigated in the present study, previous studies have shown that H_2_O_2_ can be generated through direct two‐electron reduction of oxygen by specific flavin‐dependent oxidoreductases under certain conditions, independent of obligatory O_2_^−^ intermediates [54, 55, 56]. Although the mechanism underlying *Rosasina*‐induced H_2_O_2_ accumulation remains unresolved, our findings suggest that selective disruption of antioxidant defenses is a critical initiating event. Future studies will therefore define the upstream molecular regulators of catalase inhibition and determine how this redox vulnerability orchestrates H_2_O_2_ accumulation and ferroptotic signaling.

The redox shift is coupled to hallmark features of ferroptosis, including expansion of the labile iron pool and enhanced lipid peroxidation. Transcriptional changes further reinforce this mechanism, with induction of *ACSL4, NCOA4, NOX5* and *TFRC*, and suppression of antioxidant genes. Functional interrogation implicates *NCOA4* and *NOX5* as key contributors linking ROS generation to lipid peroxidation, positioning these pathways as key drivers of *Rosasina*‐induced ferroptotic cell death. Notably, these effects are largely absent in non‐malignant cells, indicating a dependence on tumor‐specific metabolic vulnerabilities.

A major challenge for pro‐oxidant therapies is to achieve sufficient selective antitumor activity without disrupting redox homeostasis in normal tissues. Consistent with this challenge, many redox‐active agents are limited by dose‐dependent systemic toxicity and narrow therapeutic windows [57, 58]. Our findings demonstrate that *Rosasina* overcomes this limitation by selectively inducing oxidative stress in glioblastoma cells while sparing normal astrocytes. The tumor‐selective activity of *Rosasina* was further demonstrated in an orthotopic glioblastoma model, where treatment suppressed tumor growth, extended survival, and maintained normal organ architecture without evidence of systemic toxicity. These preclinical safety findings are consistent with previous toxicological and clinical evidence supporting the favorable tolerability of ClO_2_^−^‐based compounds, including early human safety studies [27, 31], regulatory toxicology assessments by The US Agency for Toxic Substances and Disease Registry (ATSDR) (https://www.atsdr.cdc.gov/toxprofiles/tp160.pdf), and a Phase II clinical trial of intravenous sodium chlorite [59], all of which reported no clinically significant treatment‐related toxicity at therapeutically relevant exposure levels. Collectively, these findings suggest that *Rosasina* selectively exploits the heightened redox vulnerability of glioblastoma while preserving physiological redox homeostasis in normal tissues, thereby providing a favorable therapeutic window for glioblastoma treatment.

Our findings also have important therapeutic implications for the current management of glioblastoma. Increasing evidence indicates that pharmacological induction of ferroptosis sensitizes glioblastoma cells to temozolomide (TMZ) by enhancing lipid peroxidation, disrupting antioxidant defenses, and overcoming adaptive resistance mechanisms [17, 18, 60]. Moreover, TMZ itself has been reported to partially activate ferroptotic signaling, suggesting that ferroptosis induction and conventional chemotherapy converge on complementary mechanisms of tumor cell death [45, 61]. In line with this, *Rosasina* suppresses the ferroptosis resistance mediators *SLC7A11* and *GPX4* while promoting oxidative stress and lipid peroxidation, thereby potentially lowering the threshold for TMZ‐induced cytotoxicity and restoring therapeutic sensitivity in resistant tumors. This therapeutic rationale is further supported by the recent identification of metabolic resistance pathways, including the PRR11–DHODH axis, which protect glioblastoma cells from ferroptotic stress and contribute to TMZ resistance [62]. By disrupting redox adaptation and reinforcing ferroptotic signaling, *Rosasina* may complement current standard‐of‐care therapies and enhance their therapeutic efficacy. Collectively, these findings provide a strong rationale for future studies to evaluate *Rosasina* in combination with current standard‐of‐care therapies, including TMZ and radiotherapy, as a strategy to overcome therapeutic resistance in glioblastoma.

Controlled redox perturbation is increasingly recognized as a critical regulator of macrophage polarization and inflammatory signaling, providing a promising therapeutic strategy for remodeling the tumor immune microenvironment [63, 64]. Beyond its direct cytotoxic effects, our findings demonstrate that *Rosasina* reprograms the glioblastoma tumor microenvironment by promoting the accumulation of M1‐like microglia/macrophages. *Rosasina* enhanced myeloid cell infiltration while selectively inducing the expression of M1‐associated mediators, including *TNF‐α*, *IFN‐γ*, *IL‐6*, *CD86*, and *STAT1*, without significantly altering M2‐associated markers, indicating a preferential shift toward a pro‐inflammatory antitumor immune phenotype. Given that tumor‐associated microglia and macrophages constitute the dominant immune population in glioblastoma, where M1‐like cells promote antitumor immunity, whereas M2‐like cells support immune evasion and tumor progression [46, 63, 64, 65], these findings suggest that *Rosasina*‐induced oxidative stress not only eliminates tumor cells directly but also functionally reprograms the myeloid compartment to enhance antitumor immunity. This dual mechanism of action highlights the therapeutic potential of *Rosasina* to simultaneously induce ferroptotic tumor cell death and reshape the immune landscape, thereby overcoming the immunosuppressive glioblastoma microenvironment and improving the efficacy of redox‐based cancer therapies.

The therapeutic benefit of ferroptosis extends beyond direct tumor cell killing by reshaping the immune microenvironment; however, the molecular mechanisms linking ferroptotic stress to macrophage polarization remain poorly defined. Here, we identify *NCOA4*‐mediated ferritinophagy and *NOX5*‐dependent oxidative signaling as critical mediators coupling *Rosasina*‐induced ferroptosis to macrophage activation. While silencing either *NCOA4* or *NOX5* had little effect on basal tumor–macrophage communication, it markedly attenuated the inflammatory M1‐like phenotype induced by *Rosasina*‐treated glioblastoma cells, demonstrating that ferroptotic signaling is required for tumor‐driven immune remodeling. These findings suggest that *Rosasina* remodels the glioblastoma secretome through *NCOA4*‐and *NOX5*‐dependent oxidative signaling, thereby promoting inflammatory macrophage polarization rather than functioning solely as a cytotoxic agent. This interpretation is supported by studies demonstrating that ferroptosis‐associated ROS activate redox‐sensitive pathways, including NF‐κB and HIF‐1α, which reinforce the metabolic and inflammatory programs underlying M1 macrophage activation [66, 67, 68, 69]. Importantly, *Rosasina* enhanced inflammatory macrophage polarization without affecting macrophage viability, indicating that these effects represent active immune reprogramming rather than nonspecific oxidative injury. These findings suggest that therapeutic activation of ferroptosis may simultaneously eliminate tumor cells and convert the glioblastoma immune microenvironment into a more immunostimulatory state, thereby improving the efficacy of future combination therapies.

Overall, our findings provide compelling preclinical evidence that *Rosasina* is a redox‐directed therapeutic capable of simultaneously inducing ferroptotic tumor cell death and remodeling the glioblastoma immune microenvironment (Figure 10). By selectively eliminating tumor cells while reprogramming macrophages toward a pro‐inflammatory phenotype, *Rosasina* targets both the intrinsic mechanisms of tumor survival and the extrinsic immunosuppressive barriers that limit current therapies. This dual mode of action, together with its favorable safety profile and robust in vivo efficacy, positions *Rosasina* as a promising candidate for translational development and supports its evaluation in combination with existing therapeutic modalities to overcome resistance and improve clinical outcomes in glioblastoma.

**FIGURE 10:**
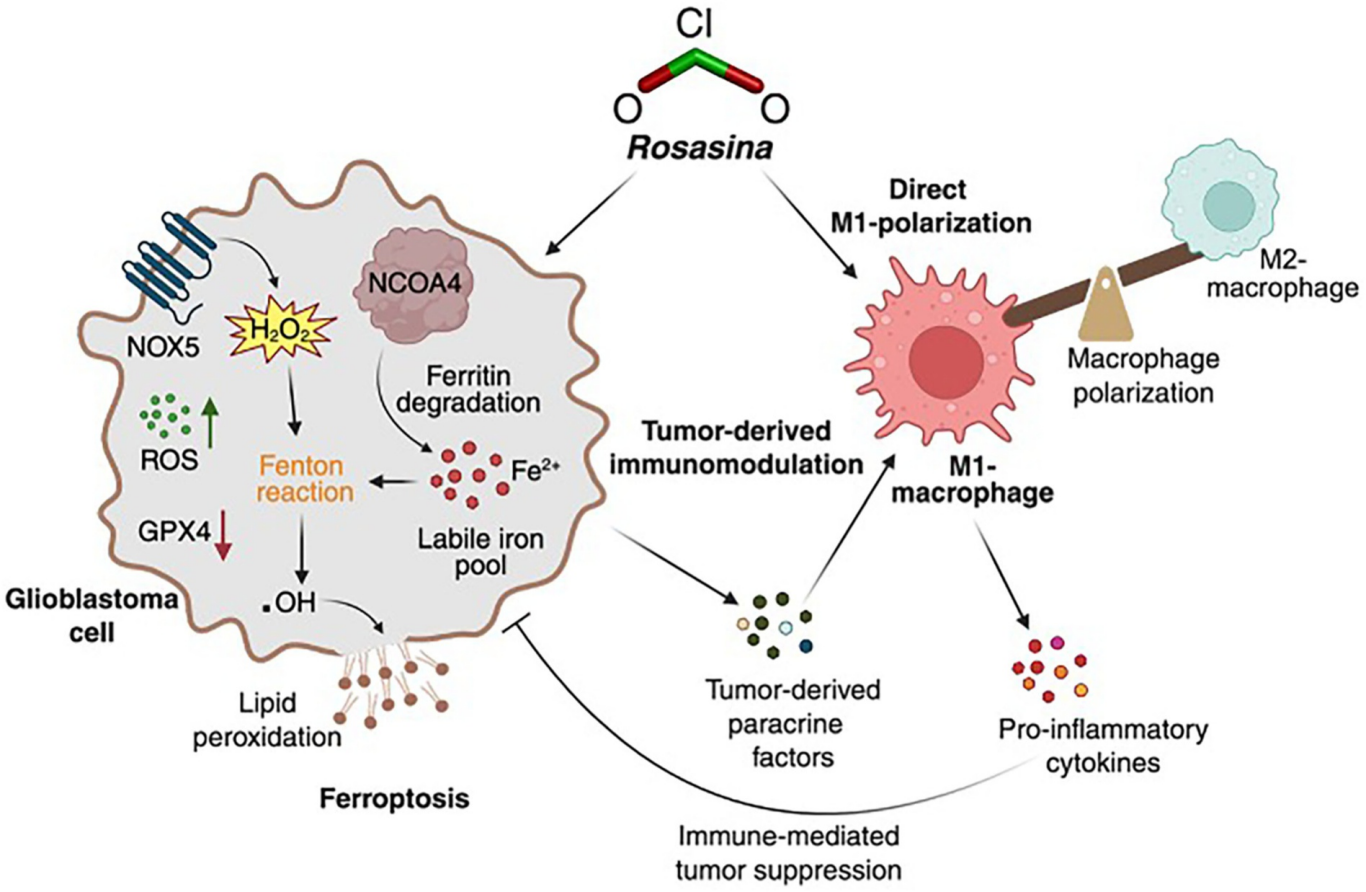
*Rosasina* mediated dual anti‐tumor activity. *Rosasina* exerts antitumor activity through the coordinated induction of ferroptosis and remodeling of the glioblastoma tumor microenvironment. *Rosasina* induces oxidative stress in glioblastoma cells by activating the *NCOA4*‐*NOX5* axis, leading to hydrogen peroxide (H_2_O_2_) production, ferritin degradation, expansion of the labile iron (Fe^2+^) pool, and enhanced Fenton chemistry. These events promote lipid peroxidation‐mediated ferroptosis of glioblastoma cells. In parallel, *Rosasina* promotes antitumor immunity through two complementary mechanisms. First, *Rosasina* directly promotes M1‐like macrophage polarization. Second, *Rosasina*‐treated glioblastoma cells release tumor‐secreted immunomodulatory factors that further stimulate M1‐like macrophage polarization. Activated M1‐like macrophages produce pro‐inflammatory cytokines, which contribute to immune‐mediated tumor suppression and reinforce the antitumor effects of *Rosasina*.

## 4 Material and Methods

### 4.1 Cell Culture

Human glioblastoma cell lines LN340, LN229, MGG123, GBM10, GBM12, GL261 and NHA were cultured as previously described [70, 71]. Briefly, the cells were cultured in Dulbecco's modified Eagle's medium (DMEM) supplemented with 10% fetal bovine serum (FBS), 2 mM L‐glutamine, and 1% penicillin‐streptomycin. In parallel, patient derived glioblastoma lines MGG123, GBM10 and 12 were grown as neurospheres in NeuroCult media (Gibco) supplemented with heparin, human epidermal growth factor (EGF), human fibroblast growth factor (FGF) and 10% fetal bovine serum (FBS) in ultra‐low attachment flasks (Nunc). All glioblastoma lines used in this study were wtIDH/umMGMT and maintained in a humidified atmosphere at 37°C incubator with 5% CO_2._ Cell line identity was verified annually by STR profiling, and cultures were routinely tested for *mycoplasma* contamination using *Mycoplasma* detection kit (ATCC).

### 4.2 Primary Human Monocyte Isolation and Differentiation

Freshly prepared buffy coats from healthy donors were obtained from the Oklahoma Blood Institute (Sylvan N. Goldman Center, Oklahoma City, OK, USA). CD14^+^ monocytes were isolated from peripheral blood mononuclear cells (PBMCs) using Ficoll‐Paque (GE Healthcare, Piscataway, NJ, USA) density‐gradient centrifugation followed by magnetic bead selection with CD14 MicroBeads (Miltenyi Biotec, Cologne, Germany) according to the manufacturer's protocol. Monocyte purity and viability exceeded 95%, as confirmed by flow cytometry.

Isolated monocytes were seeded at a density of 2 × 10^6^ cells/mL in DMEM supplemented with penicillin (100 U/mL), streptomycin (100 μg/mL), and gentamicin (50 μg/mL). After 2 h of adherence, the medium was replaced with DMEM containing 10% heat‐inactivated FBS (Life Technologies, Grand Island, NY, USA) and recombinant human GM‐CSF or M‐CSF (50 ng/mL each; PeproTech, Rocky Hill, NJ, USA) to induce M1‐or M2‐like macrophage differentiation, respectively, as previously described [72, 73, 74]. Following 7 days of differentiation, macrophages were treated with the indicated concentrations of *Rosasina* for subsequent analyses.

### 4.3 *Rosasina* Preparation

*Rosasina*, a sodium chlorite (NaClO_2_)‐derivative, was freshly prepared immediately prior to use. Equal volumes of 22% NaClO_2_ solution (126 μL) and 25% citric acid (126 μL) were combined in a microcentrifuge tube, gently mixed by pipetting, and incubated at room temperature for 30 sec. The mixture was diluted with 848 μL of distilled water to yield a final stock concentration of 93.82 μM. For experimental treatments, a 1 μM working solution (final volume 500 μL) was prepared by diluting 5.32 μL of the stock solution into 494.68 μL of distilled water.

### 4.4 Microscopy

Intracellular ROS imaging: Intracellular reactive oxygen species (ROS) levels were quantified using the 2′,7′‐dichlorofluorescein diacetate (DCFH‐DA) assay, as previously described with minor modifications [75]. Briefly, DCFH‐DA is hydrolyzed by intracellular esterases to non‐fluorescent H_2_DCF, which is subsequently oxidized by ROS to generate the highly fluorescent 2′,7′‐dichlorofluorescein (DCF). The fluorescence intensity of DCF is directly proportional to intracellular ROS levels. NHA and LN340 cells were seeded in 6‐well plates and cultured to 80%–90% confluency at 37°C in a humidified atmosphere containing 5% CO_2_. Freshly isolated, viable PBMCs (0.5 × 10^6^ cells per well), were seeded in 48‐well flat‐bottom plates and allowed to rest overnight [76, 77]. Cells were incubated with 10 μM DCFH‐DA for 30 min at 37°C in the dark, followed by treatment with *Rosasina* at the indicated concentrations for specified time points (30–240 min). After treatment, cells were washed and fluorescence was acquired using a Leica DMi8 fluorescence microscope equipped with the LAS X application suite using the FITC filter set (Ex 470/40 nm; Em 525/50 nm) at 10x magnification. All experimental groups were imaged using identical acquisition parameters.

Lipid peroxidation imaging: NHA and LN340 cells were seeded in 12‐well plates and treated with *Rosasina* (10 nM) or Ferrostatine‐1 (1 μM) (Tocris Bioscience) for 24 h. Lipid peroxidation was assessed using BODIPY 581/591 C11 (Thermo Fisher Scientific) according to the manufacturer's instructions. Briefly, BODIPY‐C11 was added directly to the culture medium, and cells were incubated for 30 min at 37°C in a 5% CO_2_ incubator. Following staining, the cells were washed three times with 1× Dulbecco's phosphate‐buffered saline (DPBS). Fluorescence was imaged using a Leica DMi8 fluorescence microscope with LAS X application suite. Oxidized BODIPY 581/591 C11 fluorescence was imaged using the FITC channel (Ex 470–490 nm/Em 500–550 nm), whereas reduced BODIPY fluorescence was imaged using the PE channel (Ex 540–560 nm/Em 580–620 nm). All experimental groups were imaged using identical acquisition parameters.

Intracellular iron (Fe^2+^) measurement: Intracellular labile iron (Fe^2+^) levels were measured using the FerroOrange fluorescent probe according to the manufacturer's instructions (Dojindo). NHA or LN340 cells were seeded in 12‐well plates and treated with *Rosasina* (10 nM) or Erastin (5 μM) (MedChemExpress) for 24 h. Following treatment, the cells were incubated with 1 μM FerroOrange solution for 30 min at 37°C. Fluorescence was acquired using a Leica DMi8 fluorescence microscope equipped with the LAS X application suite, using an excitation at 561 nm and emission between 570 and 620 nm. All experimental groups were imaged using identical acquisition parameters.

### 4.5 Flow Cytometric Analysis

Intracellular ROS measurement: After treatment (treatment conditions see intracellular ROS imaging), cells were harvested, washed twice and resuspended in 1x phosphate‐buffered saline (PBS) for analysis. ROS production was quantified using flow cytometry (BD FACS Melody) using the FITC channel. For PBMCs, immune cell subsets including CD3^+^ T cells, CD19^+^ B cells, CD14^+^ monocytes, and CD11c^+^ dendritic cells were identified using specific antibody staining and standard gating strategies. Data were analyzed using FlowJo (v10.10.0), and ROS levels were estimated as the median fluorescence intensity (MFI) of viable cells. Across all cell types, DCF fluorescence intensity directly correlated with intracellular ROS levels.

Apoptosis assay: Cells (LN340, NHA) were seeded (8x104/well) in a 12‐well plate and cultured for 24 h. The cells were exposed to *Rosasina* (1–1000 nm) or vehicle 24 h. The treated and control cells were harvested by centrifugation at 1200 rpm for 5 min, washed with 1x PBS, and then 1x Annexin‐V binding buffer (Cat# 00‐0055‐43). The processed cells were resuspended in 1x Annexin‐V binding buffer (100 μL) and incubated with Annexin V‐FITC (Cat# 11‐8005‐72, Thermo Scientific) antibody (5 μL) at 37°C for 15 min. Cells were washed with 10 volumes of 1x Annexin‐V binding buffer and resuspended in 5 μL of propidium iodide (Cat# 00‐6990‐42, Thermo Scientific) containing 1x Annexin‐V binding buffer (200 μL). The suspensions were incubated on ice for 10 min and analyzed using a Beckman coulter CytoFLEX flow cytometer (C00772, Beckman coulter, Chaska, MN). Twenty thousand events were recorded for each sample, and the data analysis was performed using FlowJo software.

Superoxide and hydrogen peroxide measurement: Intracellular superoxide (O_2_^−^) and hydrogen peroxide (H_2_O_2_) levels were quantified using a fluorescence‐based flow cytometry assay according to the manufacturers' protocols (Abcam and AAT Bioquest) with minor modifications. Briefly, cells were cultured to 50%–60% confluency in a 6‐well plate. A cell superoxide detection assay kit (ab139477) was used to stain the cells for 1 h at 37°C. The cells were treated with *Rosasina* at the indicated concentrations for specified time points (30–240 min). Pyocyanin (250 μm, 30 min) was used as a positive control to induce O_2_^−^ generation. After incubation, cells were washed and analyzed by flow cytometry using a 488 nm excitation laser.

OxiVision Blue Peroxide Sensor (AAT Bioquest, 11,505) was utilized for hydrogen peroxide (H_2_O_2_) measurement: Cells were incubated with 500 μL of the peroxide sensor solution for 30 min at 37°C, followed by the *Rosasina* treatment. Hydrogen peroxide (100 μm, 60 min) served as a positive control. After treatment, cells were washed and subjected to flow cytometric analysis using the Pacific Blue channel (Ex/Em = 405/450 nm). In both assays, fluorescence was quantified within viable cell populations, and signal intensity was expressed as median fluorescence intensity (MFI).

Cell sorting of microglia/macrophages: For tumor associated microglia/macrophage analysis, single cell suspensions were prepared from freshly harvested green fluorescent protein (GFP) expressing GL261 tumors as previously described [46] and stained with LIVE/DEAD Fixable Aqua Dead Cell Stain (Life Technologies) and primary antibodies against CD45‐Alexa700 (mouse, 30‐F11) and CD11b‐APC (mouse, M1/70). Sorting for GFP^−^CD45^+^CD11b^+^ microglia/macrophages was performed using a BD FACSAria (BD Biosciences) 11 color high‐speed sorter [46]. Flow cytometry sorted cells were subjected to RT‐PCR analysis of macrophage polarization markers.

Macrophage polarization: Cells were harvested and washed in ice‐cold PBS supplemented with 1% FBS and 0.08% sodium azide. Debris was excluded based on forward scatter (FSC) and side scatter (SSC) parameters, and doublets were eliminated using FSC/SSC pulse‐width discrimination. Monocyte/macrophage populations were identified by FSC/SSC characteristics, with the monocyte gate comprising approximately 60% of the total events. For polarization analysis, cells were stained with fluorophore‐conjugated human specific antibodies against M1 markers: IFNγ‐PE and HLA‐DR‐FITC, and M2 markers: CD163‐APC and CD206‐PE (BD Pharmingen or BioLegend, San Diego, CA, USA). Unstained and isotype‐matched controls were included to determine background fluorescence. Data acquisition was performed using a BD Accuri C6 flow cytometer (BD Biosciences, San Jose, CA, USA), and analyses were conducted using FlowJo software (Tree Star, Ashland, OR). Final gating was refined to include large, granular macrophage populations while excluding low‐scatter debris [74, 78].

### 4.6 Superoxide Dismutase Activity

Superoxide dismutase (SOD) activity was determined using the pyrogallol autoxidation assay, as previously described [79]. Briefly, 280 μL of phosphate‐EDTA buffer (pH 7.9) was added to each well of a 96‐well plate, followed by 10 μL of cell lysate and 10 μL of freshly prepared pyrogallol solution. The kinetics of pyrogallol autoxidation was monitored by measuring the increase in absorbance at 420 nm at 30 sec intervals for 5 min using a microplate reader (BioTek). SOD activity was calculated based on the inhibition of pyrogallol autoxidation relative to a buffer control.

### 4.7 Catalase Activity

Catalase activity was measured using a colorimetric hydrogen peroxide (H_2_O_2_) decomposition assay according to the manufacturer's instructions (RayBio, MA‐CAT), with minor modifications. Briefly, 20 μL of cell lysate was added to each well of a 96‐well plate, followed by 30 μL of H_2_O_2_ substrate solution (1 mM). The reaction mixture was incubated at room temperature for 1 min, terminated by the addition of catalase quencher, and absorbance was measured at 520 nm using a microplate reader (BioTek). Catalase activity was calculated based on a standard curve.

### 4.8 Cell Viability Assay

Cell viability was assessed using the MTT assay kit (ab211091). LN340 and NHA cells, as well as human, monkey, and mouse peripheral blood mononuclear cells (PBMCs) and primary human monocyte‐derived macrophages were seeded in 96‐well plates. The cells were treated with the indicated concentration of *Rosasina* for 24 h. Following treatment, 3‐(4,5‐dimethylthiazol‐2‐yl)‐2,5‐diphenyl tetrazolium bromide (MTT) reagent was added to each well and incubated for 4 h at 37°C in the dark followed by addition of dimethyl sulfoxide (DMSO). Absorbance was measured at 570 nm using a microplate reader (BioTek). Cell viability was calculated relative to untreated controls.

### 4.9 In Vivo Toxicity, Xenograft and Survival Studies

Six‐week‐old healthy male and female C57BL/6J mice were obtained from The Jackson Laboratory. Mice were housed in a specific pathogen‐free environment within an AAALAC‐accredited Research Animal Resources facility of the University of Minnesota, under veterinary supervision and in accordance with AAALAC guidelines. These animals were not involved in any prior studies. All experimental procedures were performed under protocols (2307‐41247A) approved by the Animal Care and Use Committee, University of Minnesota.

For the in vivo *Rosasina* toxicity studies, C57BL/6J mice (*n* = 6 per group, 3 male and 3 female) weighing 19–22 g were used for the study. *Rosasina* was administered orally at a dose of 2 mg/kg/day once daily for 14 or 21 consecutive days. Animals were closely monitored throughout the treatment period for general health status and behavioral changes. Body weight was recorded at baseline and monitored throughout the treatment period. At the end of the treatment period, blood samples were collected from the orbital venous plexus into capillary tubes prior to sacrifice and organs were collected. Serum was separated by centrifugation at 1000 × g for 5 min and stored at −80°C until further biochemical analysis. The biochemical parameters, including alanine aminotransferase (ALT), aspartate aminotransferase (AST), alkaline phosphatase (ALP), lactate dehydrogenase (LDH), N‐acetyl‐β‐D‐glucosaminidase (NAG), cholesterol, and blood urea nitrogen (BUN), were quantified using commercially available diagnostic kits according to the manufacturer's instructions (Cell Biolabs). After sacrifice, major organs such as liver, kidney and spleen tissue samples were collected for histopathological analysis.

C57BL/6J mice (6 weeks old; *n* = 8 per group, 4 male and 4 female or *n* = 6 per group, 3 male and 3 female) were anesthetized via intraperitoneal injection of ketamine (100 mg/kg, Dechra) and xylazine (10 mg/kg, Dechra), and anesthesia was confirmed by loss of reflexes. Following a midline scalp incision, a burr hole was created, and tumor cells were stereotactically implanted into the right striatum using a murine stereotaxic apparatus (Stoelting Co.). Luciferase‐expressing GL261 (*n* = 8) or GFP‐GL261 (*n* = 6) cells were injected at coordinates 1.8 mm lateral to the right of the bregma and 3.0 mm below the dura. Seven days after tumor implantation, mice were randomly assigned to treatment groups and received *Rosasina* (2 mg/kg/day) or vehicle by oral gavage once daily until the experimental endpoint. Animals were monitored daily for neurological deficits and general health, including body weight, lethargy, and hunched posture, and were euthanized upon reaching predefined humane endpoints. Survival was monitored for up to 45 days after tumor implantation and analyzed using Kaplan‐Meier survival curves. Statistical significance was determined using the log‐rank test.

### 4.10 Bioluminescence Imaging

Mice were anesthetized by inhalation of 2% isoflurane and the dose was maintained throughout the imaging procedures. 150 mg/kg D‐luciferin, dissolved in 1x PBS, was injected into the peritoneum, and the mice underwent sequential exposures in auto mode (IVIS50 imaging system) according to the manufacturer's instructions. The images were acquired on days 7, 14, and 21 post‐tumor implantations. Bioluminescence intensity was assessed using the Living Image 3.2 software (Caliper Life Sciences, Hopkinton, MA). Total flux values (photons (p)/second (sec)) were determined by delineating the regions of interest (ROI) of uniform size on each mouse.

### 4.11 Histology and Microscopic Analysis

Extracted tissues including liver, kidney, spleen and tumor tissues were fixed in 10% neutral buffered formalin and stored in 70% ethanol, embedded in paraffin, sectioned, mounted onto slides, and 5 μm histologic sections were subjected to routine hematoxylin and eosin (H&E) staining. Tumor tissue sections (4 μm) were placed onto positively charged glass slides. The slides were deparaffinized, rehydrated and antigen retrieval was performed. Endogenous peroxidase was blocked using 3% hydrogen peroxide followed by Dako Protein Serum Block. Primary antibody, IBA‐1 (Microglia‐1, Biocare cat# CP290B, 1:600 dilution) was incubated for 30 min at RT. Detection was achieved using Rabbit EnVision+ Kit (cat# K4003, Dako) and developed using diaminobenzidine (Dako) chromogen. Slides were counterstained with Mayer's hematoxylin.

Following staining, the slides were blindly reviewed by a board‐certified veterinary pathologist, and for each, a brief narrative description and semiquantitative assessment of microscopic findings were generated. Histological sections were evaluated and scored using a previously published semiquantitative scoring system [80] (as appropriate for each tissue type) assessing necrosis, cellular degeneration, fibrosis, neovascularization, thrombosis, inflammation, lymphoid depletion, and tissue rarefaction. Each parameter was scored as follows: 0 (absent), 1 (minimal), 2 (mild), 3 (moderate), and 4 (marked). For each Iba‐1 stained section, a total of 10 random and 400x fields were evaluated (5 random intratumoral and 5 random peritumoral fields). In each field, the density of Iba‐1 positive cells was scored as 0 (no Iba‐1 positive cells), 1 (Iba‐1 positivity in 1%–10% of nucleated cells), 2 (Iba‐1 positivity in 11%–33% of nucleated cells), 3 (Iba‐1 positivity in 34%–67% of nucleated cells), or 4 (Iba‐1 positivity in 67%–100% of nucleated cells).

### 4.12 Cell Lysate Preparation and Immunoblotting

Cells were harvested, washed twice with ice‐cold 1× PBS and resuspended in lysis buffer (20 mM Tris‐HCl (pH 7.4), 150 mM NaCl, 2 mM EDTA, and 1% NP‐40) supplemented with protease inhibitor cocktail (Sigma). The lysates were clarified by centrifugation at 14,000 × g for 5 min at 4°C. Supernatants were collected and concentrations were determined using a DC Protein Assay kit (Bio‐Rad). The protein mixtures were subjected to 4%–20% gradient SDS/PAGE electrophoresis, followed by transfer to a nitrocellulose membrane (Biorad). Membranes were blocked with 5% BSA for 1 h at room temperature and then incubated overnight at 4°C with the indicated primary antibody (*NCOA4*: Cell Signaling Technology #66849; *NOX5*: sc‐518114; SOD2: Cell Signaling Technology #13194S; CAT: Cell Signaling Technology #14097S); GAPDH (Proteintech‐10494‐1‐AP). After washing with 1x TBST buffer, membranes were incubated with horseradish peroxidase‐conjugated secondary antibodies (Cell Signaling Technology) for 1 h at room temperature and developed using chemiluminescence reagent (GE biosciences) and imaged using the Bio‐Rad ChemiDoc MP Imaging System.

### 4.13 siRNA and Plasmid Transfection, RNA Isolation and RT‐qPCR

LN340 cells were transfected with 30 nM siRNA targeting human *NCOA4* (siRNA ID# 107703) and *NOX‐5* (siRNA ID# 109551) (Thermo Fischer Scientific) using Lipofectamine RNAiMAX (Invitrogen) according to the manufacturer's instructions. For overexpression studies, LN340 cells were transfected with 2 μg of pcDNA3.1‐*NCOA4*‐FLAG or pcDNA3.1‐*NOX5*‐FLAG plasmids (OriGene) using Lipofectamine 2000 (Invitrogen) following the manufacturer's protocol.

Total RNA was isolated from cells using the miRNeasy Kit (Qiagen) following the manufacturer's instructions. cDNA was synthesized from the total RNA using Ecodry premix reverse transcription kit (Takara Bio). mRNA transcripts were quantified by qPCR using target‐specific primer sets (Supporting Information S1: Table S2) and SYBR Green (Bio‐Rad) on a Bio‐Rad Chromo4 DNA Engine real‐time PCR system.

### 4.14 Macrophage‐Glioblastoma Cell Co‐Culture

Human PBMCs were differentiated into macrophages by culturing cells in DMEM medium supplemented with 10% fetal bovine serum (FBS) and recombinant human M‐CSF (50 ng/mL) for 6 days, with media replacement every 2–3 days. LN340 glioblastoma cells were cultured and transfected with *siNT* or *siNCOA4* or *siNOX5* using Lipofectamine RNAiMAX according to the manufacturer's instructions (Invitrogen). Forty‐eight hours post‐transfection, the cells were treated with 10 nM *Rosasina* for 12 h and transferred to the upper chamber of Transwell inserts (0.4 μm pore‐size), while day‐6 M0 macrophages were plated in the lower chamber. The cells were co‐cultured for 24 h at 37°C in a humidified incubator containing 5% CO_2_. Following co‐culture, macrophages were collected from the lower chamber. Expression of macrophage polarization markers, including *TNF‐α, IFN‐γ, IL‐6, CD86, STAT1, CD163, CD206, ARG1*, *IL‐10* and *STAT6*, was normalized to *GAPDH* expression. Relative gene expression was calculated using the 2^−ΔΔ*Ct*^ method.

### 4.15 Statistical Analysis

Three or more independent experiments were performed for each assay, and the results were combined to define as mean ± SD. Statistical analysis was conducted using Microsoft Excel and GraphPad Prism software 10. The statistical significance was evaluated using an unpaired two‐tailed Student's *t*‐test or one‐way ANOVA. Following ANOVA, post hoc multiple comparisons were conducted using Tukey's or Dunnett's or Mann‐Whitney test to identify statistically significant differences between specific group pairs. *p* value (*) of ≤ 0.05 was considered statistically significant.

## Author Contributions

G.S. conceived, designed, and supervised the study. S.S., I.M., K.K., R.A.N., D.D., and L.M. performed the experiments. D.S. performed the histopathological analysis of the tissue samples. A.R.N., A.S., and S.K. contributed new reagents/analytic tools. S.S., I.M., R.A.N., D. S., A.R.N., A.S., K.B.‐L., S.K., and G.S. analyzed data. S.S. and G.S. drafted the manuscript. S.S., I.M., R.A.N., D.D., A.R.N., A.S., K.B.‐L., S.K., and G.S. critically revised the manuscript. All authors approved the final manuscript.

## Ethics Statement

All animal experiments were approved by the University of Minnesota Institutional Animal Care and Use Committee (IACUC protocol no. 2307‐41247A) and conducted in accordance with institutional guidelines. Human peripheral blood mononuclear cells (PBMCs) were obtained as deidentified samples through U Market at the University of Minnesota. Deidentified human peripheral blood mononuclear cells (PBMCs) were purchased through the U Market at the University of Minnesota. Under the University's Investigator Manual (HRP‐103), the authors determined that their use did not constitute human subjects research requiring institutional review board approval. Donor consent was managed by the supplier, and no additional informed consent was required.

## Conflicts of Interest

The authors declare no conflicts of interest.

## Supporting information


Supporting Information S1

## Data Availability

All data reported in this manuscript will be shared by the corresponding author upon request. This paper does not report the original code.
