# Supplementary material for: A Selective Pro‐Oxidant Drives Redox Reprogramming to Induce Ferroptosis and Immune Remodeling in Glioblastoma
Source: Smart Med. 2026 Oct 8;5(5):e70058. doi: 10.1002/smmd.70058 (PMC13650181; doi:10.1002/smmd.70058)
Supplement: Supplementary file 1 [file SMMD-5-e70058-s001.docx]

**A selective pro-oxidant drives redox reprogramming to induce ferroptosis and immune remodeling in glioblastoma**

Shilpi Singh^1^, Iteeshree Mohapatra^2^, Katelyn Kim^1^, Raza Ali Naqvi^3^, Devanjan Dey^4^, Laura Mohapatra^1^, Davis Seelig^5^, Afsar R. Naqvi^3^, Amar Singh^4^, Kathleen Boris-Lawrie^6^, Stefan Kim^1^, Gatikrushna Singh^1*^

^1^Department of Neurosurgery, University of Minnesota, Minneapolis, MN, 55455, USA

^2^Bimeda Biologicals, Oakdale, MN, 55128, USA

^3^Department of Periodontics, College of Dentistry, University of Illinois Chicago, Chicago, IL, 60612, USA

^4^Schulze Diabetes Institute, Department of Surgery, University of Minnesota, Minneapolis, MN, 55455, USA

^5^Department of Veterinary Clinical Sciences, College of Veterinary Medicine, University of Minnesota, St. Paul, MN, 55108, USA

^6^Department of Veterinary and Biomedical Sciences, University of Minnesota, Saint Paul, MN 55108

Running title: *Rosasina* induces dual anti-tumor activity

**^*^Corresponding author:**

**Gatikrushna Singh**, PhD, Department of Neurosurgery, University of Minnesota, 515 Delaware St. SE, Moos 2-153, Minneapolis, MN, 55455, USA, Tel.: 612-301-6042, Fax: 612-624-0644, email: gsingh@umn.edu

**Sup. Figure 1. *Rosasina* induces dose and time dependent ROS generation in immune cell populations.**

Flow cytometric analysis of reactive oxygen species (ROS) levels was performed in distinct immune cell subsets. (**A**) T cells, (**B**) dendritic cells, (**C**) monocytes, (**D**) B cells after treatment with *Rosasina* at increasing concentrations (10 nM, 100 nM, and 1000 nM) for 30 min and 240 min. Representative histograms show the fluorescence intensity of DCF-DA, indicating intracellular ROS levels, compared to untreated controls. A progressive rightward shift in fluorescence with increasing *Rosasina* concentration reflects enhanced ROS generation. Quantitative analysis of mean fluorescence intensity (MFI) is presented as bar graphs (mean ± SD, *n* = 3).

**Sup. Figure 2. *Rosasina* alter catalase enzymatic activities.**

Normal human astrocytes (NHA) and LN340 glioblastoma cells were treated with *Rosasina* at concentrations of 10 nM, 100 nM, and 1000 nM for 30 min, 120 min, and 240 min. Total cellular proteins were extracted, catalase (CAT) and superoxide dismutase (SOD) activity was determined. CAT activity was determined using a colorimetric assay based on the decomposition of hydrogen peroxide (H₂O₂). SOD activity was quantified using a pyrogallol-based colorimetric assay. Enzymatic activity was calculated as units per microgram (U/µg) of total protein. (**A-B**) NHA, (**C-D**) LN340, (**E-F**) PBMCs. * *p* <0.05, *** *p* <0.001 between untreated and *Rosasina* treated groups (Student's t-test).

**Sup. Figure 3. *Rosasina* alters expression of CAT and SOD glioblastoma cells.**

LN340 glioblastoma cells were treated with *Rosasina* at concentrations of 10 nM for 24 h. Total RNA was and protein was extracted. The RNA was processed for cDNA preparation by RT-PCR. Catalase (CAT) and superoxide dismutase (SOD) expression was determined by qPCR. (**A**) CAT, (**B**) SOD. Total protein was used for immunoblotting of catalase (**C**) and SOD (**D**). *** *p* <0.001 between untreated and *Rosasina* treated groups (Student's t-test).

**Sup. Figure 4. ROSASINA does not alter body weight in treated mice.**

C57BL/6J mice (*n*=6: 3 females and 3 males per group) were treated with *Rosasina* (2 mg/kg/day) or H₂O vehicle control for 14 or 21 days. Body weight was recorded every other day and plotted over time. (**A**) Body weight during treatment up to day 14. (**B**) Body weight during treatment up to day 21.

**Sup. Figure 5. Semi-quantitative histopathological assessment of liver, kidney, and spleen.**

Liver, kidney, and spleen from vehicle- and *Rosasina*-treated mice were collected at the experimental endpoint, fixed in 10% neutral-buffered formalin, paraffin-embedded, sectioned (5 μm), and stained with hematoxylin and eosin (H&E). H&E-stained sections were evaluated in a blinded manner by a board-certified veterinary pathologist for tissue-specific microscopic abnormalities, including necrosis, degeneration, fibrosis, neovascularization, thrombosis, inflammation, lymphoid depletion, and rarefaction, as applicable (indicated in the plot for each tissue analysis). Histopathological changes were scored using a semi-quantitative scale: 0: absent, 1: minimal, 2: mild, 3: moderate, and 4: marked. Data are presented as mean ± SD. **(A)** Liver; **(B)** Kidney; **(C)** Spleen.

**Sup. Figure 6. Systemic safety and histopathological evaluation of the liver, kidney, and spleen in tumor-bearing mice treated with *Rosasina.***

Liver, kidney, and spleen from vehicle- and *Rosasina*-treated GL261 tumor-bearing mice were collected at the experimental endpoint, fixed in 10% neutral-buffered formalin, paraffin-embedded, sectioned (5 μm), and stained with hematoxylin and eosin (H&E). H&E-stained sections were evaluated in a blinded manner by a board-certified veterinary pathologist for tissue-specific microscopic abnormalities, including necrosis, degeneration, fibrosis, neovascularization, thrombosis, inflammation, lymphoid depletion, and rarefaction, as applicable (indicated in the plot for each tissue analysis). Histopathological changes were scored using a semiquantitative scale: 0: absent, 1: minimal, 2: mild, 3: moderate, and 4: marked. Data are presented as mean ± SD. **(A)** Liver; **(B)** Kidney; **(C)** Spleen.

**Sup. Figure 7. *Rosasina* did not induce apoptosis in glioblastoma cells.**

Normal human astrocytes (NHA) and LN340 glioblastoma cells were treated with *Rosasina* at 10, 100, and 1,000 nM for 30, 120, and 240 min. Cells were subsequently stained with Annexin V–FITC and propidium iodide (PI) and analyzed by flow cytometry. Representative scatter plots show the distribution of viable (Annexin V⁻/PI⁻), early apoptotic (Annexin V⁺/PI⁻), late apoptotic (Annexin V⁺/PI⁺), and necrotic (Annexin V⁻/PI⁺) cell populations in NHA and LN340 cells.

**Sup. Figure 8. NCOA4 and NOX5 as critical mediators of *Rosasina*-induced ferroptosis.**

(**A**) Western blot validation of siRNA-mediated knockdown of *NCOA4* and *NOX5* in LN340 cells. Cells were transfected with *siNT*, s*iNCOA4*, or *siNOX5*. Forty-eight hours post-transfection, cell lysates were harvested and Western blot for indicated antibodies. (**B**) Overexpression of FLAG tagged NCOA4 and NOX5 in LN340 cells. Forty-eight hours post-transfection of FLAG tagged *NCOA4* and *NOX5* plasmids, cell lysates were prepared and Western blot for FLAG antibody. (**C**) Cell viability assessment of ectopic expression of NCOA4 and NOX5 in LN340 cells treated with or without *Rosasina* (10 nM) for 24 h. Cell viability was determined using a standard MTT assay. ****p*<0.001; *****p*<0.0001. (**D**) Fluorescent detection of lipid peroxidation using BODIPY-C11 probe in NCOA4, and NOX5 over expressing LN340 cells with or without *Rosasina*. Red and green fluorescence denote oxidized and reduced BODIPY forms, respectively; merged images depict oxidative status at the single image.

**Table S1. Organ weight measurement post-*Rosasina* treatment.**

|  | **Liver** | **Kidney** | **Spleen** | **Heart** | **Lung** | **Brain** |  |
| --- | --- | --- | --- | --- | --- | --- | --- |
|  |  |  | **Day14** |  |  |  |  |
| Vehicle | 1.075 ± 0.10 | 0.3 ± 0.08 | 0.1 ± 0.0 | 0.15 ± 0.06 | 0.2 ± 0 | 0.4 ± 0.0 |  |
| *Rosasina* | 1.075 ± 0.15 | 0.275 ± 0.1 | 0.1 ± 0.0 | 0.125 ± 0.05 | 0.15 ± 0.06 | 0.4 ± 0.0 |  |
| **Day 21** | | | | | | | |
| Vehicle | 1.08 ± 0.15 | 0.3 ± 0.00 | 0.1 ± 0.0 | 0.1 ± 0.0 | 0.15 ± 0.06 | 0.4 ± 0.0 |  |
| *Rosasina* | 1.175 ± 0.13 | 0.35 ± 0.06 | 0.1 ± 0.0 | 0.1 ± 0.0 | 0.2 ± 0.00 | 0.4 ± 0.0 |  |

**Table S2. Oligonucleotide sequences of primers used in the study.**

| **Gene** | **Sequence** |
| --- | --- |
| NOX-5-F | 5’ GGATCAACAGAGACCAGC 3’ |
| NOX-5-R | 5’ CCCAGTGCAGATGTCATGTAC 3’ |
| ACSL4-F | 5’ AAGTAGACCAACGCCTTCAG 3’ |
| ACSL4-R | 5’ GTCCCAGTCCAGGTATTCTTTC 3’ |
| ALOX-15-F | 5’ GCCGTCGATACATCCTATCTTC 3’ |
| ALOX-15-R | 5’ TGGCCCGGACGTTAATTTC 3’ |
| SCD1-F | 5’ GACCTAGAAGCTGAGAAACTGG 3’ |
| SCD1-R | 5’ GCAAGAAAGTGGCAACGAAC 3’ |
| ACSL3-F | 5’ AAGTTCGAAGCTTGCTAGGG 3’ |
| ACSL3-R | 5’ CAGATTCAGTGAGCCCGTATC 3’ |
| PTGS2-F | 5’ TGTATGAGTGTGGGATTTGACC 3’ |
| PTGS2-R | 5’ TGTGTTTGGAGTGGGTTTCAG 3’ |
| TFRC-F | 5’ TCTCCTATTGAAACTTGCCCAG 3’ |
| TFRC-R | 5’ GTATCCCTCTAGCCATTCAGTG 3’ |
| NCOA4-F | 5’ GAATACCTTCCAAGACCAGAGTG 3’ |
| NCOA4-R | 5’ AGCTTTGACCTCTCGCAAG 3’ |
| FTH1-F | 5’ AGAGGGAACATGCTGAGAAAC 3’ |
| FTH1-R | 5’ CACACTCCATTGCATTCAGC 3’ |
| SLC40A1-F | 5’ CTACTTGTGCCTCCCAGATG 3’ |
| SLC40A1-R | 5’ GCCCAGGACAGTCATATAAAGG 3’ |
| SLC7A11-F | 5’ TTTTGTACGAGTCTGGGTGG 3’ |
| SLC7A11-R | 5’ CGCAAGTTCAGGGATTTCAC 3’ |
| GPX4-F | 5’ ATCCACGAATGTCCCAAGTC 3’ |
| GPX4-R | 5’ CCGGTACTTGTCCAGGTTAAC 3’ |
| AIFM2-F | 5’ GTCACTCTCATTCACTCCCAAG 3’ |
| AIFM2-R | 5’ GATCTCCTTCACTTCCTGCC 3’ |
| GCH1-F | 5’ AAGACTACAAGTTCAGGAGCG 3’ |
| GCH1-R | 5’ ACACCTCGCATTACCATACAC 3’ |
| NRF2-F | 5’ GTTCCTGATATTCCCGGTCAC 3’ |
| NRF2-R | 5’ CCAAACTTGCTCAATGTCCTG 3’ |
| LPCAT3-F | 5’ TGCTTCAGTTCCTCATCCTTC 3’ |
| LPCAT3-R | 5’ CGGTGGCAGTGTAATAGTATCC 3’ |
| SOD-F | 5’ TGCAGGTCCTCACTTTAATCC 3’ |
| SOD-R | 5’ AAGTCTCCAACATGCCTCTC 3’ |
| CAT-F | 5’ GGAGATTCAACACTGCCAATG 3’ |
| CAT-R | 5’ TCTGTTCCTCATTCAGCACG 3’ |
| IFN-γ-F | 5’ CAGCAACAGCAAGGCGAAAAAGG 3’ |
| IFN-γ-R | 5’ TTTCCGCTTCCTGAGGCTGGAT 3’ |
| TNF-α-F | 5’ CAGAAACACAAGATGCTGG 3’ |
| TNF-α-R | 5’ CAAAAGAGGAGGCAACAAGG 3’ |
| IL-6-F | 5’ TCTGCAAGAGACTTCCATCC 3’ |
| IL-6-R | 5’ TTAGCCACTCCTTCTGTGAC 3’ |
| CD86-F | 5’ CCATCAGCTTGTCTGTTTCATTCC 3’ |
| CD86-R | 5’GCTGTAATCCAAGGAATGTGGTC 3’ |
| STAT-1-F | 5’ ATGGCAGTCTGGCGGCTGAATT 3’ |
| STAT-1-R | 5’ CCAAACCAGGCTGGCACAATTG 3’ |
| CD206-F | 5’ GTTCAGCTATTGGACGCGAG 3’ |
| CD206-R | 5’ GAATCTGACACCCAGCGGAA 3’ |
| ARG1-F | 5’AACACGGCAGTGGCTTTAACC 3’ |
| ARG1-R | 5’ GGTTTTCATCTGGCGCATTC 3’ |
| IL10-F | 5’ CCTTAATGCAGGACTTTAAGGGTTA 3’ |
| IL10-R | 5’ TTACACTCGCCCCCTTTGC 3’ |
| STAT-6-F | 5’ CCTTGGAGAACAGCATTCCTGG 3’ |
| STAT-6-R | 5’ GCACTTCTCCTCTGTGACAGAC 3’ |
| GAPDH-F | 5’ ACCCAGAAGACTGTGGATGG 3’ |
| GAPDH-R | 5’ TTCTAGACGGCAGGTCAGGT 3’ |
| α-tubulin-F | 5’ ACTCTGATTGTGCCTTCATGG 3’ |
| α-tubulin-R | 5’ AATCTCAGGGAAGCAGTGATG 3’ |
| β-actin-F | 5’ GGAAATCGTGCGTGAC 3’ |
| β-actin-R | 5’ ATGCCCAGGAAGGAA 3’ |
